# Brassinosteroid overproduction improves lignocellulose quantity and quality to maximize bioethanol yield under green-like biomass process in transgenic poplar

**DOI:** 10.1186/s13068-020-1652-z

**Published:** 2020-01-18

**Authors:** Chunfen Fan, Hua Yu, Shifei Qin, Yongli Li, Aftab Alam, Changzhen Xu, Di Fan, Qingwei Zhang, Yanting Wang, Wanbin Zhu, Liangcai Peng, Keming Luo

**Affiliations:** 1 Chongqing Key Laboratory of Plant Resource Conservation and Germplasm Innovation, Key Laboratory of Eco-environments of Three Gorges Reservoir Region, Ministry of Education, Institute of Resources Botany, School of Life Sciences, Southwest University, Chongqing, 400715 China; 2 Biomass & Bioenergy Research Centre, College of Plant Science & Technology, Huazhong Agricultural University, Wuhan, 430070 China; 3 College of Biomass Sciences and Engineering, College of Agronomy and Biotechnology, China Agricultural University, Beijing, 100193 China

**Keywords:** *Populus*, Brassinosteroid, Bioethanol, Saccharification, Xylem differentiation, Lignocellulose modification, Green-like pretreatment

## Abstract

**Background:**

As a leading biomass feedstock, poplar plants provide enormous lignocellulose resource convertible for biofuels and bio-chemicals. However, lignocellulose recalcitrance particularly in wood plants, basically causes a costly bioethanol production unacceptable for commercial marketing with potential secondary pollution to the environment. Therefore, it becomes important to reduce lignocellulose recalcitrance by genetic modification of plant cell walls, and meanwhile to establish advanced biomass process technology in woody plants. Brassinosteroids, plant-specific steroid hormones, are considered to participate in plant growth and development for biomass production, but little has been reported about brassinosteroids roles in plant cell wall assembly and modification. In this study, we generated transgenic poplar plant that overexpressed *DEETIOLATED2* gene for brassinosteroids overproduction. We then detected cell wall feature alteration and examined biomass enzymatic saccharification for bioethanol production under various chemical pretreatments.

**Results:**

Compared with wild type, the *PtoDET2* overexpressed transgenic plants contained much higher brassinosteroids levels. The transgenic poplar also exhibited significantly enhanced plant growth rate and biomass yield by increasing xylem development and cell wall polymer deposition. Meanwhile, the transgenic plants showed significantly improved lignocellulose features such as reduced cellulose crystalline index and degree of polymerization values and decreased hemicellulose xylose/arabinose ratio for raised biomass porosity and accessibility, which led to integrated enhancement on biomass enzymatic saccharification and bioethanol yield under various chemical pretreatments. In contrast, the CRISPR/Cas9-generated mutation of *PtoDET2* showed significantly lower brassinosteroids level for reduced biomass saccharification and bioethanol yield, compared to the wild type. Notably, the optimal green-like pretreatment could even achieve the highest bioethanol yield by effective lignin extraction in the transgenic plant. Hence, this study proposed a mechanistic model elucidating how brassinosteroid regulates cell wall modification for reduced lignocellulose recalcitrance and increased biomass porosity and accessibility for high bioethanol production.

**Conclusions:**

This study has demonstrated a powerful strategy to enhance cellulosic bioethanol production by regulating brassinosteroid biosynthesis for reducing lignocellulose recalcitrance in the transgenic poplar plants. It has also provided a green-like process for biomass pretreatment and enzymatic saccharification in poplar and beyond.

## Background

As the main components of agricultural and forestry waste, lignocellulose represents enormous biomass resource for biofuels and biochemical production [1, 2]. Although agricultural residues and dedicated energy crops provide a large amount of lignocellulose for cellulosic ethanol production, woody biomass (softwood and hardwood) cannot be ignored to be a very important feedstock, for example, woody biomass occupies approximately 30% of total biomass in the US [3]. Woody biomass has nearly no ash and lower content of pentoses [4], which reduces transportation and processing cost and is conducive to the bioconversion to produce ethanol, and woody biomass can be harvested all year round to make long-term storage unnecessary [4, 5]. However, compared with grasses, woody plants have higher lignin content because of their growth behavior of becoming physically larger and stronger. This makes woody biomass, particularly that from softwood species, more recalcitrant to microbial and enzymatic hydrolysis, further leading to an unacceptable cost for using woody biomass as biofuels [6]. Therefore, overcoming the recalcitrance of woody biomass is promising in biofuel production.

Lignocellulose recalcitrance is fundamentally determined by plant cell wall composition, cell wall polymer features and cell wall structure [7, 8]. Plant cell walls are composed mainly of cellulose, hemicelluloses, and lignin. Cellulose consists of *β*-1, 4-glucan chains that form microfibrils with crystalline and amorphous regions. Cellulose crystalline index (CrI) and degree of polymerization (DP) are well demonstrated as the key negative factors accounting for biomass enzymatic digestibility [9–11]. In contrast, the arabinose (Ara) substitution degree of hemicelluloses in grasses has positive influence on biomass enzymatic saccharification by reducing the cellulose crystallinity [11, 12]. Lignin typically has a negative impact on biomass enzymatic digestibility under various chemical pretreatments. However, recent findings have suggested that lignin could enhance biomass yield and lignocellulose enzymatic digestion [12, 13]. Lignin is thus considered to play dual roles in lignocellulose digestion, depending on distinct monomer proportions. The non-specific binding of cellulases to lignin negatively impacts process economics through deactivating enzyme activities during hydrolysis [13, 14]. Additionally, biomass porosity and cellulose accessibility act as the potentially positive factors accounting for biomass saccharification of lignocellulose residues after chemical pretreatments [15, 16]. Hence, genetic improvement of lignocellulose features could lead to significantly enhanced enzymatic saccharification of biomass and bioethanol yield [8, 16–20].

For cellulosic ethanol production, initial biomass pretreatments are considered as crucial step for enhancing sequential enzymatic hydrolysis and final yeast fermentation [1, 21–23]. Over the past years, various chemical pretreatments have been performed to reduce lignocellulose recalcitrance in grasses, such as H_2_SO_4_, NaOH, CaO, Na_2_S + Na_2_CO_3_ [24–29]. Acids and alkalis (H_2_SO_4_ and NaOH) are the classical agents applied for pretreatment to enhance biomass enzymatic hydrolysis in woody plants, but these methods release wastes and cause serious secondary environmental pollution. Therefore, it is essential to find out an optimal biomass process technology for efficient enzymatic hydrolysis with less secondary pollution release in woody plants.

In woody plants, xylem is the main component of stem. Xylem development is a complex process controlled by a network for the coordinating regulation of several diverse metabolic pathways [30]. Brassinosteroids (BRs), plant-specific steroid hormones, are considered to participate in xylem development [31]. Initially, the BRs are biosynthesized from campesterol (CR) via the early C-6 and late C-6 oxidation pathways. For the early C-6 oxidation pathway, campestanol (CN) is converted to 6-oxocampestanol (6-oxoCN) and then to cathasterone (CT), teasterone (TE), 3-dehydroteaserone (3DT), typhasterol (TY), and castasterone (CS), respectively. In the late C-6 oxidation pathway, CN is chiefly to form 6-deoxocathasterone (6-deoxoCT) and then converted to corresponding intermediates similar to those in the early C-6 oxidation pathway, but in a C-6 deoxy forms. A CN-independent pathway, C-22 oxidation branch, is demonstrated to occur alongside the previously reported CR to CN pathway, and suggested to be the dominant upstream BR biosynthesis pathway [32]. In these pathways, a series of enzymes have been characterized: DWARF4 (DWF4), CONSTITUTIVE PHOTOMORPHOGENESIS AND DWARFISM (CPD), DEETIOLATED2 (DET2), ROTUNDFOLIA3 (ROT3/CYP), and BR-6-oxidase1 (BR6ox1/2) [32]. During the enzymatic steps, DET2 catalyzes a 5α-reduction of multiple related sterols, and is an important rate-limiting enzyme in the BR biosynthesis pathway [32]. BRs are perceived at the plasma membrane by the receptor-like kinase BRASSINOSTEROID INSENSITIVE1 (BRI1) and ASSOCIATED-KINASE1 (BAK1). BRs binding result in the dissociation of a negative regulator, BRI1 KINASE INHIBITOR 1 (BKI1), to disassociate from BRI1, and initiate a phosphorylation cascade of BRASSINOSTEROID INSENSITIVE 2 (BIN2) kinase. BIN2 controls the stabilization and activation of BRASSINAZOLE-RESISTANT1 (BRZ1) and BR-INSENSITIVE-EMS-SUPPRESSOR 1 (BES1/BZR2), and therefore to regulate the transcription of BR-responsive target genes [31, 32]. Overexpression of BR biosynthesis or signaling genes led to more xylem formation and increased cell wall deposition [31–36]. Although increasing evidence has established the connection between BRs and wood formation, the role of BRs during cell wall polysaccharide biosynthesis and modification is not well revealed, and the effects of BRs in bioethanol production are largely unknown.

Poplar is a fast-growing and widely distributed tree species, providing most woody materials around the world. In this study, we isolated *PtoDET2* from *Populus tomentosa* Carr., characterized its role in xylem and cell wall formation during wood development, and evaluated its effect on biomass enzymatic saccharification and bioethanol production. Meanwhile, the major cell wall polysaccharide features and biomass porosity were determined. By comparing various chemical pretreatments, we find out an optimal technique relatively economical and environment-friendly for high bioethanol production. This study also proposed a mechanism model interpreting why higher bioethanol yield was achieved in the transgenic poplar under pretreatments.

## Results

### *PtoDET2* overexpression improved plant growth and biomass yield in poplar

**Fig. 1:**
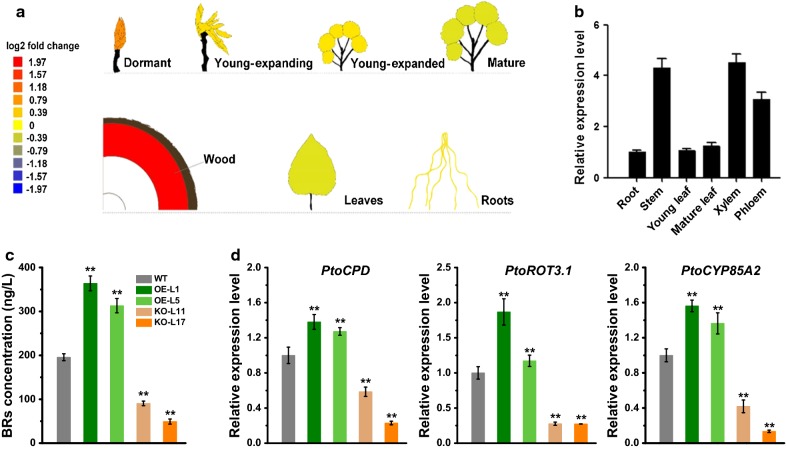
Collection of *PtoDET2* transgenic poplars. **a** *PtoDET2* expression in different developmental stages throughout the most periods of life cycle in poplar. **b** *PtoDET2* expression profiling by Q-PCR analysis. **c** Endogenous BRs levels in transgenic and wild-type stems. **d** The expression of BRs biosynthesis relative genes. Data represent mean ± SD of three biological replicates. Statistical analyses were performed using Student’s *t* test as **P < 0.01

The full-length coding sequence of *PtoDET2* (Potri.016G110600.1) was cloned from cDNA of *P. tomentosa* using sequence-specific primers (Additional file 1: Tables S1 and S2). Multiple sequence alignment revealed that PtoDET2 shared high identity with PtrDET2 (97.67%) in *P. trichocarpa*, PeDET2 (96.89%) in *P. euphratica* (Additional file 1: Fig. S1). Using public expression profiling data (http://aspwood.popgenie.org), we found that *PtoDET2* is mainly expressed in wood tissues, while accumulated lower in buds, leaves, and roots (Fig. 1a). To verify this, we analyzed *PtoDET2* expression in developing organs of *P. tomentosa* by quantitative PCR (Q-PCR). As expected, *PtoDET2* was expressed strongly in the secondary cell wall-forming zone of stems, such as xylem and phloem (Fig. 1b), suggesting that *PtoDET2* was tightly associated with wood development in poplar.

To investigate the function of *PtoDET2* in poplar growth and development, we collected the *PtoDET2*-overexpressed (OE) and -knockout (KO) mutant lines using the CRISPR/Cas9-based genome editing system. Five independent *PtoDET2*-OE lines were obtained with relatively higher *PtoDET2* gene expression levels, compared to the WT (Additional file 1: Fig. S2A–C). The phenotypes of OE lines showed a significantly positive correlation with the *PtoDET2* gene expression levels, in which, OE-L1, L5 were regenerated based on higher *PtoDET2* expression levels. More than 10 putative *PtoDET2*-KO transgenic plants were generated and sequenced. Two *PtoDET2* loss-of-function mutants (L11, L17), which were translational frame-shift or premature termination with insertions and deletions in three sgRNA-targeted sites, were regenerated for further analysis (Additional file 1: Fig. S2D–E). All the regenerated transgenic lines (more than 10 plans for each line) exhibited consistently phenotypes with the primary generation.

Because the *PtoDET2* encodes an essential enzyme involved in brassinosteroids (BRs) biosynthesis, this study determined endogenous BRs contents. As a result, BRs levels were significantly elevated in the stems of *PtoDET2*-OE transgenic poplars, while decreased in *PtoDET2*-KO lines at *P* < 0.01 level, compared with control (Fig. 1c). Meanwhile, we detected three major genes (*PtoCPD*, *PtoROT3*, *PtoCYP85A2*) expression, which are responding to the BRs biosynthesis in the downstream steps [33], and all three genes were significantly up-regulated in *PtoDET2*-OE lines, but down-regulated in *PtoDET*-KO lines (Fig. 1d), consistent with the altered BRs levels.

**Fig. 2:**
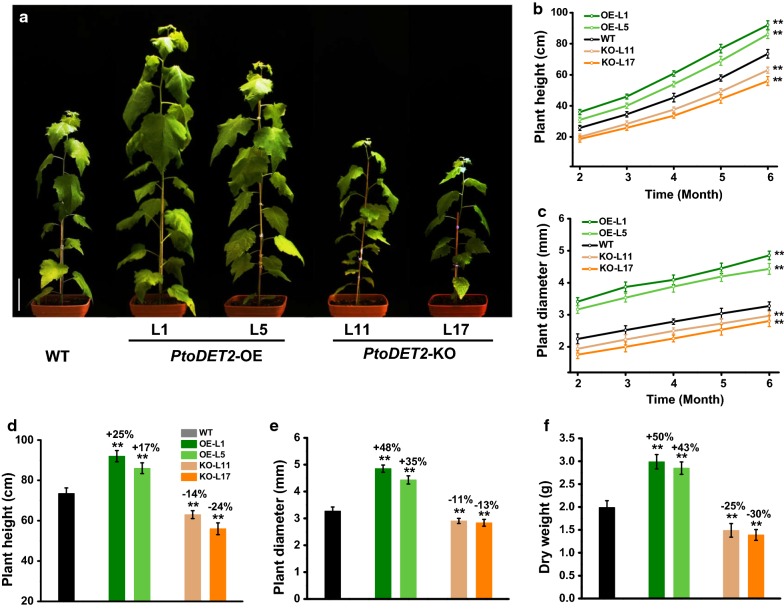
Measurement of plant growth and biomass yield in transgenic poplar plant. **a** Images of 5-month-old transgenic poplar lines and wild type (WT); Scale bar as 10 cm. **b**, **c** Observation of plant growth in the transgenic lines and WT during time course of 6 months. **d**–**f** Plant height, stem diameter and dry weight (biomass yield) in the transgenic lines and WT of 6-month-old. Data represent mean ± SD of five biological replicates. Student’s *t* test was performed between the transgenic lines and WT as **P < 0.01

BRs are the plant-specific steroid hormones that dynamically regulate plant growth and development [30–36]. In this study, we observed a fast growth and development with more internodes and larger leaves in the *PtoDET2*-OE transgenic lines (Fig. 2a). Notably, during the 6-month period of growth, the *PtoDET2*-OE lines maintained much enhanced growth compared to the WT, including increased plant height by 17–25% and raised stem diameter by 35–48%, leading to total aerial dry biomass elevated by 43–50% (Fig. 2b–f). In addition, the *PtoDET2*-OE-L1 line showed a faster growth than that of the L5 line, consistent with *PtoDET2* expression levels and BRs contents in these lines. Inversely, transgenic *PtoDET2*-KO lines displayed retarded growth and lower dry biomass compared with that of WT plants (Fig. 2). Hence, our results indicated that *PtoDET2* could significantly increase BRs level for much enhanced plant growth and biomass yield, consistent with the previous findings that BRs-related genes could promote plant growth [31–40].

### *PtoDET2* affected wood formation and wall polysaccharides biosynthesis

**Fig. 3:**
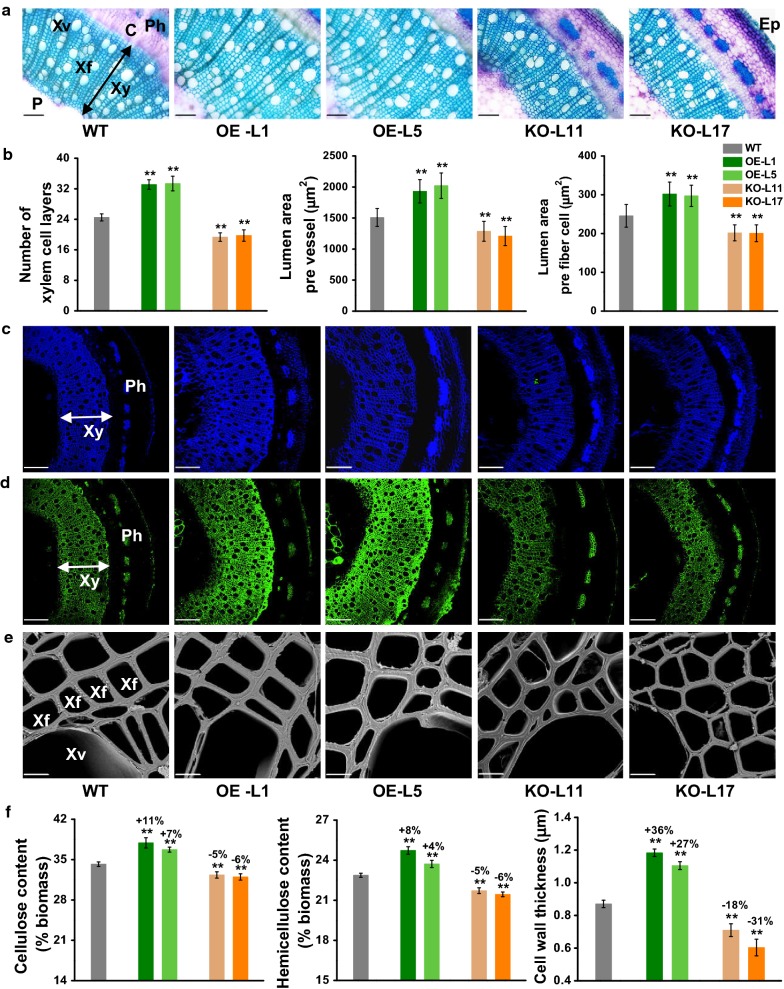
Observations of plant cell walls in transgenic poplar plant. **a** Toluidine blue staining of the 6th internode stems of 5-month-old transgenic line and WT (Ph: phloem, C: cambium, Xy: xylem, Xf: xylem fiber cells, P: pith, Ep: epidermis. Scale bars as 50 μm). **b** Numbers of xylem cell layers and lumen area of individual xylem vessel cell and fiber cell. **c** Calcofluor staining specific for glucans (scale bars as 100 μm). **d** Immunohistochemical fluorescence (green) specific for xylan using LM10 antibody (scale bars as 100 μm). **e** Scanning electron microscopy (SEM) images (Xv: xylem vessel, scale bars as 5 μm). **f** Cell wall composition and cell wall thickness of SEM observation. All data as mean ± SD. Student’s *t* test was performed between the transgenic line and WT as **P < 0.01 (*n* = 3 for cell wall composition, *n* = 30 for cell wall thickness, technical replicates)

With respect to the changed biomass yield in the *PtoDET2* transgenic plants as described above, this study observed stem morphology and determined cell wall composition in the transgenic plants and WT (Fig. 3). Compared to the WT, the *PtoDET2*-OE transgenic lines exhibited much expanded xylem area with significantly increased stem diameter and amounts of xylem cell layers as well as larger vessel/fiber cells (Fig. 3a, b), in agreement with the previous reports that BRs promote differentiation of xylem cells [33–35]. Accordingly, we detected significantly increased transcription levels of four representative genes involved in xylem cell differentiation and expansion (Additional file 1: Fig. S3A–D), in supporting for the enhancement of xylem formation and cell size (Fig. 3a, b) in *PtoDET2*-OE plants.

As plant cell walls represent major components of biomass, we measured cell wall composition of the transgenic plants. By comparison, the two *PtoDET2*-OE transgenic lines contained significantly more cellulose and hemicelluloses levels than those of the WT at *P* < 0.01 levels (Fig. 3c–f), but with a similar lignin content (Additional file 1: Table S3). Using Calcofluor white staining for mixed-linkage glucans and monoclonal antibody (LM10) specific for xylan, we observed relatively stronger fluorescent signals in xylem tissues of the *PtoDET2*-OE lines (Fig. 3c, d), and detected drastically up-regulated genes involved in cellulose and hemicelluloses biosynthesis (Additional file 1: Fig. S3C, D), correspondingly for increased celluloses and hemicelluloses levels in the *PtoDET2*-OE lines. Furthermore, we observed thickened cell walls in xylem tissues of *PtoDET2*-OE transgenic lines (Fig. 3e), and measured significantly increased cell wall width by 27–36% relative to WT plants (Fig. 3f). Therefore, these data demonstrated that the *PtoDET2*-OE plants were of significantly increased biomass due to higher cellulose and hemicelluloses levels, and thicker cell wall relative to WT plants. In contrast, reduced xylem development, cell wall disposition and wall thickness were also observed in *PtoDET2*-KO plants (Fig. 3).

### *PtoDET2* enhanced biomass saccharification in transgenic poplar after chemical pretreatments

**Fig. 4:**
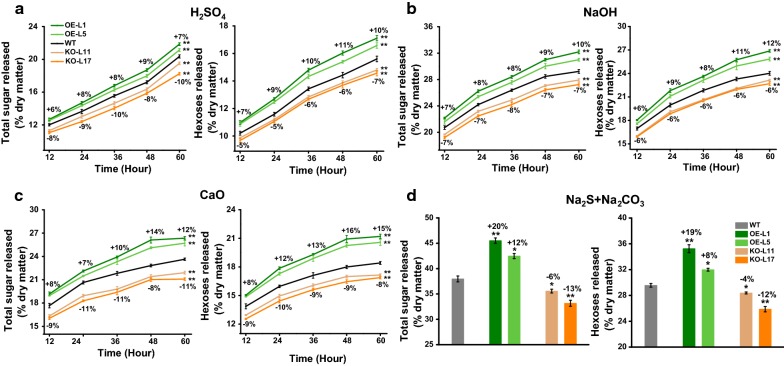
Analyses of biomass enzymatic saccharification in the transgenic lines and WT. **a** Total sugar yields and hexose yields released from enzymatic hydrolysis after the pretreatment with 4% H_2_SO_4_, **b** 4% NaOH, **c** 10% CaO or **d** Na_2_S + Na_2_CO_3_ pretreatments. Data represent mean ± SD of three technical replicates. All data as mean ± SD. Student’s *t* test between transgenic line and WT as **P < 0.01

Based on the chemical pretreatments with grassy biomass established in our previous works [15–17], here we performed various acid and alkali pretreatments with poplar biomass residues (WT) using a series of concentrations of H_2_SO_4_, NaOH, CaO and green liquor (Na_2_S + Na_2_CO_3_) under different incubation temperatures, and then determined biomass saccharification by calculating hexose yields (% biomass) released from enzymatic hydrolysis of pretreated biomass residues (Additional file 1: Fig. S4). Hence, four optimal acid and alkali pretreatments (H_2_SO_4_/120 °C/20 min; 4% NaOH/50 °C/2 h; 10% CaO/50 °C/2 h; and Na_2_S + Na_2_CO_3_/150 °C/20 min;) were established subjective to the relatively high hexose yields achieved in poplar. Using the four optimal pretreatments, this study compared biomass saccharification between transgenic poplar lines and WT. In general, two *PtoDET2*-OE lines remained significantly increased biomass saccharification including hexoses and total sugar yields (Fig. 4). By comparison, *PtoDET2*-KO transgenic lines had reduced total sugar and hexoses yields. In particular, the green liquor (Na_2_S + Na_2_CO_3_) pretreatment caused the most increased total sugar and hexoses yields by 12–20% and 8–19% in the *PtoDET2*-OE lines relative to WT. Furthermore, the Na_2_S + Na_2_CO_3_ pretreatment led to the highest hexoses and total sugar yields in both transgenic lines and WT, among the four optimal chemical pretreatments, indicating that the Na_2_S + Na_2_CO_3_ pretreatment should be most effective for enhancing biomass enzymatic saccharification in poplar.

### Improved bioethanol yield and sugar–ethanol conversion rates in *PtoDET2*-OE lines

**Fig. 5:**
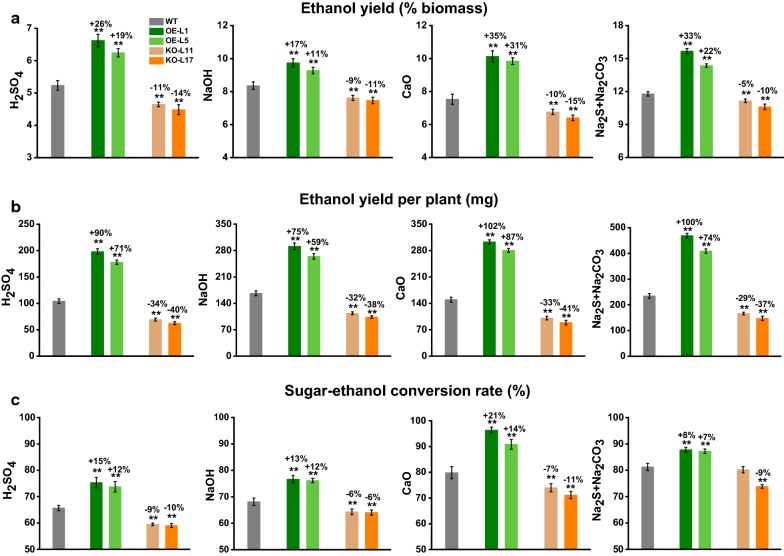
Detection of bioethanol yield and sugar–ethanol conversion rate in the transgenic lines and WT. **a** Bioethanol yields (% biomass) or **b** bioethanol yields (per plant) obtained from yeast fermentation using total hexose contents released from enzymatic hydrolysis after pretreatments. **c** Sugar–ethanol conversion rates under pretreatments. Data represent mean ± SD of three technical replicates Student’s *t* test was performed between the transgenic line and WT as **P < 0.01

To investigate bioethanol yield and sugar–ethanol conversion rates of transgenic plants, we further performed a classic yeast fermentation using total hexoses released from enzymatic hydrolysis of the pretreated poplar biomass residues. Under four optimal chemical pretreatments, two *PtoDET2*-OE lines showed significantly higher bioethanol yields (% biomass) than those of the WT at *P* < 0.01 level. In detail, the pretreatment with H_2_SO_4_ or NaOH could lead to bioethanol yields 19–26% or 11–17% increased from *PtoDET2*-OE lines than WT, whereas CaO and Na_2_S + Na_2_CO_3_ pretreatments released about 31–35% or 22–33% more bioethanol, compared with WT (Fig. 5a). Since the biomass production was improved in *PtoDET2*-OE plants, we also accessed the bioethanol yield per plant (mg). Consequently, the *PtoDET2*-OE plants had increased bioethanol yields by 71–90%, 59–75%, 87–102%, and 74–100%, after H_2_SO_4_, NaOH, CaO and Na_2_S + Na_2_CO_3_ pretreatments, respectively (Fig. 5b). Hence, these results suggested that the *PtoDET2*-OE plants were of consistently enhanced bioethanol production.

During the pretreatment process, a range of sugar and lignin degradation products are produced, such as weak acids, furan derivatives, and phenolic compounds, which have been widely reported as inhibitors of ethanol fermentation. The sugar–ethanol conversion rates were used to assess the critical levels for inhibition of ethanol production by yeast. Compared with WT, the *PtoDET2*-OE lines revealed significantly improved sugar–ethanol conversion rates under multiple pretreatments (Fig. 5c), while transgenic *PtoDET2*-KO lines showed reduced ethanol yields and conversion rates (Fig. 5). The results confirmed that *PtoDET2*-OE lines should release less amounts of toxic byproducts that inhibit yeast fermentation, probably due to its altered cell wall composition and wall polymer features.

### Altered lignocellulose features in transgenic poplar plants

**Fig. 6:**
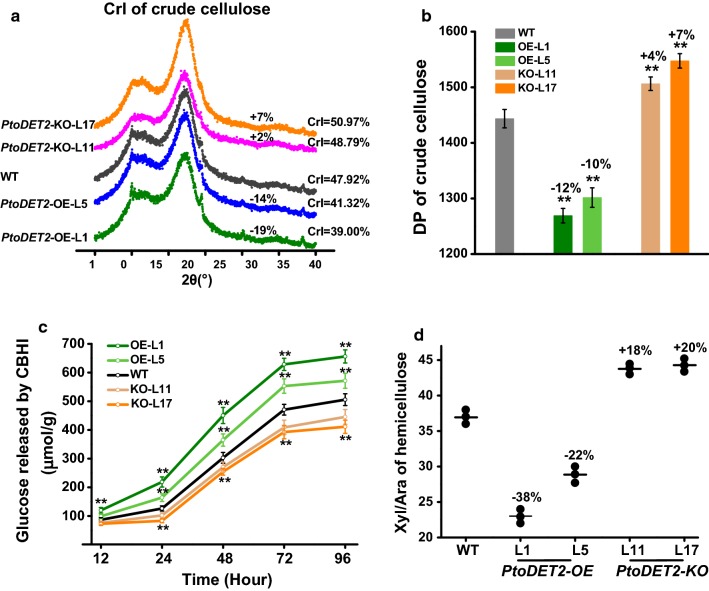
Comparison of lignocellulose features between the transgenic lines and WT. **a** Crystalline index (CrI) of crude cellulose. **b** Degree of polymerization (DP) of crude cellulose. **c** Glucose yield of the cellobiose released from time-course CBHI hydrolyzes using crude cellulose as substrate. **d** Xyl/Ara rate of total hemicelluloses. All data as mean ± SD of three technical replicates; increased percentage (%) obtained by subtracting transgenic line value with WT divided by WT. Student’s *t* test was performed between the transgenic line and WT as **P < 0.01

It has been demonstrated that lignocellulose features significantly affect biomass enzymatic saccharification under various physical and chemical pretreatments [21–29]. Due to markedly increased cellulose levels in *PtoDET2*-OE lines, we examined cellulose crystalline index (CrI) and degree of polymerization (DP), which have been examined as major cellulose features negatively affecting biomass enzymatic saccharification. Compared to WT, the *PtoDET2*-OE lines showed significantly reduced CrI and DP values by 14–19% and 10–12%, respectively (Fig. 6a, b). Previous studies show that *GH9* genes encoded glycoside hydrolase enzymes play important roles in reducing cellulose CrI and DP [18, 19, 41–43]. We found that transcript levels of two representative *PtoGH9*s were significantly increased in the *PtoDET2*-OE lines relative to WT (Additional file 1: Fig. S5), supporting the findings of reduced cellulose CrI and DP. As cellobiohydrolase (CBH) enzyme is specific for attacking the reducing ends of *β*-1, 4-glucan chains [19], we also performed an enzymatic hydrolysis in vitro using commercial CBHI enzyme (E.C. 3.2.1.91). During the time course of enzymatic hydrolysis, the *PtoDET2*-OE lines remained to release much more glucose than that of the WT (Fig. 6c), consistent with the previous reports that the reduced cellulose DP could lead to much increased glucose release from CBHI hydrolysis [19].

Meanwhile, we also determined monosaccharide composition of hemicellulose in transgenic plants and WT (Additional file 1: Table S4). By comparison, two *PtoDET2*-OE lines showed significantly increased arabinose, with reduced xylose/arabinose (Xyl/Ara) ratios than those of the WT (Fig. 6d). The Xyl/Ara ratio of hemicelluloses has been known as the negative factor on biomass enzymatic saccharification in the grassy biomass residues examined [12, 17, 19, 31]. Therefore, we speculate that the significant reduction in the Xyl/Ara ratio might be an additive reason for enhanced biomass saccharification in *PtoDET2*-OE poplar plants, though the hemicelluloses of grasses and dicots are quite different. In contrast, *PtoDET2*-KO transgenic lines showed increased CrI, DP, CBHI activity and Xyl/Ara ratio. In addition, this study detected a similar monolignol constituent of lignin between transgenic lines and WT (Additional file 1: Table S4), suggesting that lignin biosynthesis is not altered in all *PtoDET2* transgenic poplar plants.

### Increased lignocellulose porosity and accessibility in *PtoDET2*-OE lines

**Fig. 7:**
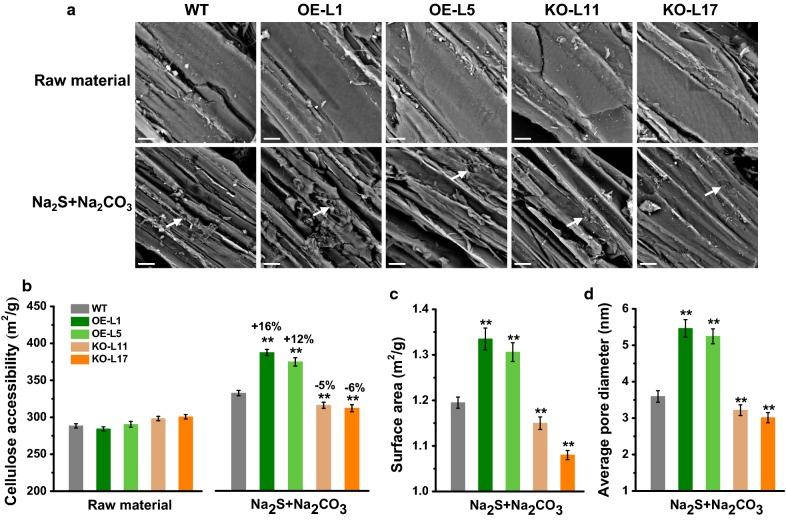
Characterization of biomass porosity and cellulose accessibility in the transgenic lines and WT. **a** SEM images of raw material and biomass residue obtained from green liquor (Na_2_S + Na_2_CO_3_) pretreatment. Scale bar is 10 μm. Allows as rough point. **b** Cellulose accessibility by measuring Congo red (CR) dye area. **c**, **d** Surface area and average pore diameter of biomass residues obtained from green liquor (Na_2_S + Na_2_CO_3_) pretreatment. All data as mean ± SD of three technical replicates. Student’s *t* test was performed between the transgenic plants and WT as **P < 0.01

Although lignocellulose features could largely affect biomass enzymatic saccharification for bioethanol production as described above, it has been recently shown that biomass porosity and cellulose accessibility of the pretreated residues are the finalized determinant of biomass enzymatic hydrolysis for bioethanol production [15, 17, 18, 42–45]. Given that the Na_2_S + Na_2_CO_3_ pretreatment is superior to other pretreatments for higher sugar and bioethanol yields in this study, the residues of Na_2_S + Na_2_CO_3_ pretreatment were used for subsequent enzyme accessibility measurement. We further observed the pretreated biomass residues by scanning electron microscopy (SEM). Compared to the raw materials, the biomass residues obtained from Na_2_S + Na_2_CO_3_ pretreatment exhibited rougher surface in both *PtoDET2* transgenic poplar and WT (Fig. 7a), consistent with the previous reports of the rough biomass residues effective for cellulase enzyme loading and digestion [15, 17, 18, 42–45]. Using the Congo-red staining approach established recently [43], we measured cellulose accessibility, which was the direct parameter of cellulase enzyme attack on cellulose surface. Despite the similar Congo-red staining areas in raw materials of both transgenic lines and WT, the pretreated residues of *PtoDET2*-OE lines showed significantly (*P* < 0.01) increased cellulose accessibility than that of WT, with the increased rate of 12–16%, while the decreased rate of 5–6% in the *PtoDET2*-KO lines (Fig. 7b), indicating that the Na_2_S + Na_2_CO_3_ pretreatment was more effective in enhancing cellulose accessibility in the *PtoDET2*-OE plants.

Since biomass porosity has been defined to account for cellulase enzyme loading and access to cellulose microfibrils [15, 17, 18, 42–45], we also detected biomass porosity of the pretreated residues including surface area and average pore diameter. By comparison, the biomass porosity of *PtoDET2*-OE lines remained significantly raised than that of the WT at *P* < 0.01 level, whereas the *PtoDET2*-KO lines showed markedly reduced biomass porosity (Fig. 7c, d). The results indicated that increased biomass porosity might result in enhanced biomass enzymatic saccharification in the *PtoDET2*-OE poplar plants.

## Discussion

Extensive evidences have shown that plant hormones are indispensable in plant growth and development. Recently, BRs were shown to take part in regulating wood formation, xylem differentiation, and cell wall biosynthesis [33–36]. Previous studies have demonstrated that DET2 catalyzes a 5α-reduction of multiple related sterols, and DET2 is an important rate-limiting enzyme in the BR biosynthesis pathway [46, 47]. In this study, overexpression of *PtoDET2* promoted plant growth and biomass yield, while knockout of *PtoDET2* led to a reduced growth (Fig. 2). These phenotypic alterations are consistent with previous studies overexpressing *PtoDWF4* and *PtCYP85A3* in poplar, which displayed increased stem height and diameter [33, 35]. All these similar phenotypes are probably due to the altered activity of the BR biosynthesis enzyme, thus influencing the levels for endogenous BRs. Hence, in this study BRs might act as a positive regulator in regulating cell number, cell size, cell wall thickness, and cell wall polymers composition in xylem (Fig. 2), finally leading to relatively higher biomass yields in *PtoDET2*-OE and correspondingly less biomass yields in *PtoDET2*-KO plants (Fig. 2). In terms of much altered cellulose and hemicelluloses levels in transgenic lines, this study also examined the major lignocellulose features, such as cellulose crystalline index (CrI), degree of polymerization (DP), and hemicellulose compositions. Compared to WT, the *PtoDET2*-OE lines showed significantly reduced CrI and DP values (Fig. 6a, b), as well as reduced Xyl/Ara ratios (Fig. 6d). Inversely, *PtoDET2*-KO lines showed increased CrI, DP, and Xyl/Ara ratios (Fig. 6). Taken together, these data implied that *PtoDET2* is positively regulates xylem development, cell wall biosynthesis and modification in poplar. It is reported that BRs promote cellulose biosynthesis and biomass accumulation in *Arabidopsis*, and BR-activated transcription factor BES1 can associate with upstream elements of most cellulose synthase genes (CESA) [48]. In this study, the expression of genes involved in cell differentiation (*LBD38*, *CLE14*), cell expansion (*EXP5*, *EXP12*), cell wall biosynthesis (*CESA*, *GT*) and glycoside hydrolase enzymes genes (*GH9*) were altered (Additional file 1: Fig. S3). It would be worthwhile to investigate whether the BES1 can bind to the promoter regions of those genes in poplar.

Besides the cellulose and hemicellulose mentioned above, lignin also has effects on biomass enzymatic hydrolysis. In plant cell wall, lignin is a phenolic polymer composed mainly of *p*-coumaryl alcohol, coniferyl alcohol and sinapyl alcohol, and the three monomers are cross-linked by ether-, ester- and C–C bonds to form a stable and water-proofing lignin complex [49, 50]. Typically, lignin has a negative impact on biomass digestibility: preventing cellulose microfibril swelling to reduce surface area access of cellulase enzymes, and limiting cellulase action on the cellulose surface [8, 13, 14]. In this study, the Na_2_S + Na_2_CO_3_ pretreatment led to the highest biomass saccharification and bioethanol yields among the four pretreatments (Figs. 4 and 5). To understand the biomass process for biofuel production under Na_2_S + Na_2_CO_3_ pretreatment, an analysis of mass balance was performed (Additional file 1: Fig. S6). Although the *PtoDET2*-OE lines had significantly higher levels of both cellulose and hemicelluloses than WT (Fig. 3f), small amounts of hexoses and pentoses were extracted in both transgenic lines and WT through the Na_2_S + Na_2_CO_3_ pretreatment (Additional file 1: Fig. S6), suggesting that the Na_2_S + Na_2_CO_3_ pretreatment is effective to restore cellulose and hemicellulose for sequential enzymatic hydrolysis and final bioethanol production. Under Na_2_S + Na_2_CO_3_ pretreatment, lignin was predominant in the extraction of both transgenic poplar lines and WT. We further observed potential alteration of wall polymer linkage resulting from the Na_2_S + Na_2_CO_3_ pretreatment using Fourier transform infrared (FTIR) spectroscopy (Additional file 1: Table S5 and Fig. S7). Compared to raw materials, the Na_2_S + Na_2_CO_3_ pretreated biomass residues showed apparent variations of four major peaks (1244, 1508, 1615, 1736 cm^−1^), which were characteristic for lignin interaction with wall networks in both transgenic lines and WT (Additional file 1: Table S5 and Fig. S7). For instance, the absorption bands located at 1736 cm^−1^ (C=O) and 1244 cm^−1^ (C–O–C) could not be found in the pretreated residues, which were referred as either ester-linked acetyl and uronic groups of hemicelluloses or the carboxylic acid groups of ferulic and *p*-coumaric acids of lignin and hemicelluloses [51]. The FTIR profiling confirmed that the Na_2_S + Na_2_CO_3_ pretreatment was also effective for lignin extraction in woody plants, consistent with the previous reports in grasses [13, 25, 27]. Despite the transgenic lines and WT had a similar lignin content in the raw materials (Additional file 1: Table S3), after Na_2_S + Na_2_CO_3_ pretreatment, 70.1 g lignin was extracted in the *PtoDET2*-OE line, but only 43.7 g and 42.3 g lignin were extracted in the WT and *PtoDET2*-KO line, respectively (Additional file 1: Fig. S6), indicating that the Na_2_S + Na_2_CO_3_ pretreatment is more effective for lignin extraction in the *PtoDET2*-OE line, probably due to its altered lignocellulose features and the weaker cross link between lignin and other wall polysaccharides. The more effective for lignin extraction in *PtoDET2*-OE lines is likely to reduce the non-specific binding of cellulases to lignin, leading to more cellulase enzymes action on the cellulose surface.

Furthermore, this study detected enzyme accessibility of raw materials and pretreated residues. As a result, the biomass residues obtained from Na_2_S + Na_2_CO_3_ pretreatment exhibited much rougher surface and increased enzyme accessibility in both transgenic poplar and WT, compared to the raw materials. This indicates that a high proportion of lignin was extracted from Na_2_S + Na_2_CO_3_ pretreatment, which largely increased biomass porosity and cellulose accessibility for cellulases access and loading, leading to remarkably enhanced biomass enzymatic saccharification and higher ethanol yield. In terms of sugar–ethanol conversion rates, the Na_2_S + Na_2_CO_3_ pretreatment caused much higher sugar–ethanol conversion rates than those of the other two optimal chemical (H_2_SO_4_, NaOH) pretreatments (Fig. 5c), confirming that this pretreatment should release less byproducts that inhibit yeast fermentation. Thereby, Na_2_S + Na_2_CO_3_ pretreatment is a relatively high effective pretreatment with lower amounts of toxic byproducts that inhibit yeast fermentation and less corrosive compounds that damage the pretreatment equipment or cause environmental pollution.

In addition, this study compared bioethanol yields (% biomass) based on the previously reported ones in three major woody plants from different biomass pretreatments (Additional file 1: Table S6). Under the Na_2_S + Na_2_CO_3_ pretreatment, the best transgenic poplar line (*PtoDET2*-OE-L1) exhibited the highest bioethanol yield of 15.68% among all reported bioethanol ones in three woody plants even though under relatively stronger pretreatment conditions [52–56]. Importantly, the bioethanol yield of our transgenic poplar (L1) line was also higher than that of the previous transgenic poplar line (*GAUT4*-KD and *MOMT4*-OE) as recently reported [52, 53]. However, both wild types (poplar) of this work and the previous study showed a similar bioethanol yield [53], suggesting that DET2 is an effective gene target for production of high-yielding biomass with improved properties for lignocellulosic biofuel production, and the Na_2_S + Na_2_CO_3_ pretreatment is a relatively highly effective pretreatment.

Based on all findings obtained in this study, we proposed a mechanistic model (Additional file 1: Fig. S8) to explain why the BRs-overproduced transgenic poplar plants have much increased biomass saccharification for the higher bioethanol production after Na_2_S + Na_2_CO_3_ pretreatment as shown below. (1) BRs regulated the expression of genes involved in cell differentiation, cell expansion, and cell wall biosynthesis (Additional file 1: Fig. S3), which results in increased xylem cell number, cell size, cell wall thickness, and cell wall polymers composition (Fig. 3), finally leading to relatively 50% higher biomass yields in *PtoDET2*-OE (Fig. 2). The cellulose and hemicelluloses, rather than lignin, were much more deposited in the thickened cell walls of *PtoDET2*-OE lines, leading to significantly high hexose/glucose yield for ethanol fermentation (Figs. 3, 4, 5). (2) Recent findings indicate that lignocellulose CrI, DP, and hemicellulosic Xyl/Ara were mainly negative factors for biomass enzymatic saccharification. In this study, the *PtoDET2*-OE lines had reduced cellulose CrI and DP, and hemicellulosic Xyl/Ara (Fig. 6), which led to an integrated positive impact on biomass enzymatic saccharification. (3) The optimal Na_2_S + Na_2_CO_3_ pretreatment is more effective for lignin extraction (Additional file 1: Figs. S6 and S7), which reduced the non-specific binding of cellulases to lignin, leading to more cellulase enzymes action on the cellulose surface. (4) The reduced cellulose CrI, DP, hemicellulosic Xyl/Ara, and the relatively high proportion of lignin extraction largely increased biomass porosity and cellulose accessibility for cellulases access and loading (Fig. 7, Additional file 1: Fig. S8), leading to remarkably enhanced biomass enzymatic saccharification and high sugar–ethanol conversion allowed to maximize bioethanol yield in the *PtoDET2*-OE plants.

## Conclusion

The present study generated BRs levels improved transgenic poplar by overexpression of the *PtoDET2*, a brassinosteroids biosynthesis gene. The BRs-improved plants exhibited increased xylem development and cell wall polymer deposition, resulting in largely enhanced biomass yield. Importantly, the *PtoDET2*-OE poplar showed significantly improved lignocellulose features such as reduced cellulose CrI, DP, hemicellulose Xyl/Ara ratio, and increased biomass porosity and accessibility, leading to remarkably enhanced biomass enzymatic saccharification and bioethanol yield. Moreover, this study compared four chemical pretreatments, and took the Na_2_S + Na_2_CO_3_ as an optimal one for efficient enzymatic hydrolysis with less secondary pollution release. Hence, this study provides a potent strategy for high cellulosic ethanol production by regulating brassinosteroids biosynthesis, and green-like biomass process for woody plants and beyond.

## Materials and methods

### Experimental procedures

#### Collection of transgenic plants

The sequences of *DET2* were obtained from JGI (https://phytozome.jgi.doe.gov/pz/portal.html), and aligned using ClustalW program implemented in MEGA7 with 1000 bootstrap replicates. The phylogenetic tree was constructed by MEGA7 with neighbor-joining (NJ) method. The GenBank accession numbers for genes used in this study are listed in Additional file 1: Table S1.

The full-length *PtoDET2* cDNA was cloned from *Populus tomentosa*, verified by sequencing, and inserted into the plant binary vector pCXSN under the control of 35S promoter. Three CRISPR/Cas9 target sites of *PtoDET2* were assembled into binary pYLCRIPSR/Cas9 vector based on their GC abundance screened in the online tool ZiFiT TARGETER v.4.2 (http://zifit.partners.org/ZiFiT/Introduction.aspx) [57]. The constructs were introduced into *Agrobacterium tumefaciens* strain EHA105 and transferred into *P. tomentosa* by *Agrobacterium*-mediated transformation as described previously [58]. The transgenic lines were selected based on the hygromycin selection and PCR analysis. To identify CRISPR/Cas9-mediated mutation of *PtoDET2* in transgenic poplar plants, the *PtoDET2* genomic fragment was cloned into the pMD19-T vector (Takara) and at least 20 clones for each transgenic line were randomly selected for sequencing. All primers used are listed in Additional file 1: Table S2.

#### Plant growth conditions and sample isolation

*P. tomentosa Carr.* plants were grown in a greenhouse under conditions of 16/8 h light/dark cycle with the 4500 lx supplementary light at 22–25 °C and relative humidity ~ 60%. Poplar plants were watered according to the evapotranspiration demands during different growth stages and fertilized with 1/2 strength Hoagland nutrient solutions. Measurement of plant growth was carried out on 10 plants for each WT and transgenic line, deleted 2–3 maximum and 2–3 minimum values, finally calculated the average values of the remained 5 plants. For biomass saccharification and cell wall composition analyses, around ~ 10 cm of the bottom part of the stems from 5 plants were harvested and the bark were peeled. The peeled stem samples were air-dried, the pith removed, the remaining tissues milled to a particle size of 20 mesh (0.85 mm), and the ground samples used for analyses.

#### Measurement of BR content

The first elongating internodes of 1-month-old soil *PtoDET2*-OE, and wild-type plants were used to measure the content of BRs. Samples of tissue powder were homogenized in PBS (PH7.2–7.4, 0.1 M). The supernatants were collected and assayed by Plant Brassinolide (BR) ELISA Kit (Beijing Chenglin Biotechnology Company, China).

#### Chemical pretreatment and biomass enzymatic hydrolysis

Dried and milled poplar was used for analysis of sugar yield. Chemical pretreatment and sequential enzymatic hydrolysis were performed as described previously with minor modifications [16, 59]. For H_2_SO_4_ pretreatment: the well-mixed biomass samples were treated with 6 mL H_2_SO_4_ under various concentrations (0%, 1%, 2%, 4%, 8% v/v) at 120 °C for 20 min, then shaken under 150 rpm at 50 °C for 2 h. For NaOH pretreatment: the well-mixed biomass samples were incubated with 6 mL NaOH under various concentrations (0%, 1%, 2%, 4%, 8% w/v) shaken at 50 °C for 2 h. For CaO pretreatment, the well-mixed biomass samples were treated with CaO at various concentrations (0%, 2%, 5%, 10%, 20% w/w) shaken at 50 °C for 48 h. For Na_2_S + Na_2_CO_3_ pretreatment, solution was prepared by mixing Na_2_S and Na_2_CO_3_ with a sulfidity (percent ratio of Na_2_S to the sum of Na_2_S and Na_2_CO_3_ on Na_2_O basis) of 30%. The samples were first impregnated with Na_2_S + Na_2_CO_3_ solution at 60 °C for 30 min. Then temperature was raised at the rate of 3 °C/min to the target temperature (130‒170 °C) and maintained for scheduled time (20 min). After pretreatments, the pretreated residues were washed with distilled water for 3‒5 times until pH 7.0 for following enzymatic hydrolysis.

Enzymatic hydrolysis: the pretreated biomass residues were washed with mixed-cellulase reaction buffer (0.2 M acetic acid–sodium acetate, pH 4.8), then incubated with 6 mL (1.6 g/L) of mixed-cellulases (Imperial Jade Biotechnology Co., Ltd. Ningxia 750002, China) co-supplied with 1% Tween-80. The sealed samples were shaken under 150 rpm for 60 h at 50 °C. After centrifugation at 3000*g* for 5 min, the supernatants were collected for pentoses and hexoses assay. All experiments were performed using five representative plants in triplicate.

#### Yeast fermentation and ethanol measurement

The yeast fermentation was conducted using *Saccharomyces cerevisiae* strain (Angel yeast Co., Ltd., Yichang, China) as previously described by Fan et al. [16] with minor modification. The activated yeast (dissolved in 0.2 M phosphate buffer, pH 4.8) was inoculated into the mixture of enzymatic hydrolysates and residues with initial cell mass concentration at 0.5 g/L. The fermentation experiments were performed at 37 °C for 48 h, and distilled for determination of ethanol content. Ethanol content was measured using the dichromate oxidation method. All experiments were performed using five representative plants in triplicate.

#### Plant cell wall fractionation and determination

Plant cell wall fractionation and assay method were conducted as described previously by Peng et al. [60] with minor modification, all experiments were performed in the technical triplicates. After removal of soluble sugars, lipid, starch and pectin from consecutive extractions with phosphate buffer (pH 7.0), chloroform–methanol, dimethyl sulphoxide/water, and 0.5% (w/v) ammonium oxalate. The crude cell walls were further suspended with the 4 M KOH containing NaBH_4_ (1.0 mg/mL), and washed three times with distilled water, the combined supernatants (KOH and distilled water) were used as KOH-extractable hemicelluloses fraction. The remaining pellets were dissolved in 72% H_2_SO_4_ (w/w) for 1 h at 25 °C, and after centrifugation, the supernatants were collected to determine hexose as cellulose level. Total hemicelluloses were calculated by measuring hexose and pentose of the hemicellulose fraction and the pentose of the remained cellulose pellets.

GC–MS analysis (SHIMADZU GCMS-QP2010 Plus) was performed for monosaccharide composition detection of hemicellulose as previously described by Fan et al. [61]. GC–MS analytical conditions: Restek Rxi-5ms, 30 m × 0.25 mm ID × 0.25 um df column. Carrier gas: He. Injection method: split. Injection port: 250 °C, interface: 250 °C. Injection volume: 1.0 μL. The temperature program: from 170 °C (held for 12 min) to 220 °C (held for 8 min) at 3 °C/min. Ion source temperature: 200 °C, ACQ Mode: SIM. The mass spectrometer was operated in the EI mode with ionization energy of 70 eV. Mass spectra were acquired with full scans based on the temperature program from 50 to 500 m/z in 0.45 s.

Total lignin content includes acid-insoluble and -soluble lignin was determined by two-step acid hydrolysis method as described [62]. The crude cell wall samples were hydrolyzed with 67% H_2_SO_4_ (v/v) at 25 °C for 90 min with a gentle shaking at 150 rpm, and subsequently diluted to 3.97% (w/w) with distilled water and heated at 115 °C for 60 min. The acid-soluble lignin was solubilized during the hydrolysis process, and was measured by UV spectroscopy at 205 nm. The remaining residues were placed in a muffle furnace at 575 ± 25 °C for 4 h for the acid-insoluble lignin assay. Lyophilized extractive-free material was used for lignin derived monomers analysis. The thioacidolysis method [63] was used to determine lignin composition. G and S lignin was isolated and quantified by GC–MS using a Hewlett-Packard 5890 series II gas chromatograph with a 5971 series mass selective detector (column: HP-1, 60 m × 0.25 mm, 0.25 μm film thickness). Mass spectra were recorded in electron impact mode (70 eV), and the scanning range was 60–650 mz^−1^.

FTIR spectroscopy was performed to observe the chemical linkages in the raw and pretreated samples using a PerkinElmer spectrophotometer (NEXUS 470, Thermo Fisher Scientific, Waltham, MA, USA), as described by Cheng et al. [50]. The well-dried biomasses were finely powdered to reduce scattering losses and deformations in the absorption band. The samples (2–4 mg) were dispersed in KBr at a weight ratio of 1:100 and subsequently pressed to produce a transparent pelletized disc by applying 1 MPa pressure for at least 2 min. The pelletized disc samples were positioned in the path of IR light and the spectra were recorded in absorption mode over 32 scans at a resolution of 4 cm^−1^ in the range of 4000 to 400 cm^−1^.

#### Detection of cellulose features (CrI, DP)

The lignocellulose crystalline index (CrI) was detected with crude cell wall materials as described by Fan et al. [16]. Essentially, crystalline cellulose was extracted using 4 M KOH (containing 1.0 mg/mL sodium borohydride) followed by 8% (w/v) NaClO_2_ with 1.5% acetic acid at 25 °C for 72 h. The pellet was washed to neutral and dried before examination with X-ray diffraction (XRD) using Rigaku-D/MAX instrument (Ultima III, Japan). The biomass powder was laid on the glass sample holder (35 × 50 × 5 mm) and detected under plateau conditions. Ni-filtered Cu Kα radiation (λ = 0.154056 nm) generated at voltage of 40 kV and current of 18 mA, and scanned at speed of 0.0197°/s from 10 to 45°. The CrI was estimated using the intensity of the 200 peak (*I*_200_, *θ *= 22.5°) and the intensity at the minimum between the 200 and 110 peaks (*I*_am_, *θ *= 18.5°) as the follow: CrI = 100 × (*I*_200_ − *I*_am_)/*I*_200_. Standard error of the CrI method was detected using five representative samples in triplicate.

The crude cellulose DP assay was performed using viscosity method as previously described according to the equation: DP^0.905^ = 0.75 [η] [10]. And [η] is the intrinsic viscosity of the solution. All experiments were performed at 25 ± 0.5 C using an Ubbelohde viscosity meter and cupriethylenediamine hydroxide (Cuen) as the solvent. The intrinsic viscosity was calculated by interpolation using the USP table (USP, 2002) that lists the predetermined values of the product of intrinsic viscosity and concentration. The [η] for cellulose samples exhibiting relative viscosity (*η*_re_) values between 1.1 and 9.9. *η*_rel_, was calculated using the equation: *η*_rel_ = *t*/*t*_0_, where t and t_0_ are the efflux times for the cellulose solution and Cuen (blank) solvent, respectively. Standard error of the DP method was detected using five representative samples in triplicate.

#### Microscopic observation

The sixth internode of the 5-month-old poplar stems were fixed in FAA buffer (formaldehyde:glacial acetic acid: 50% ethanol, 1:1:18). After embedding in paraffin, the stems were cross sectioned by using an Ultra-Thin Semiautomatic Microtome (FINESSE 325, Thermo) and stained with 0.05% (w/v) toluidine blue O and then observed under Zeiss optical microscope (Zeiss, Oberkochen, Germany).

For cellulose staining, sections were incubated with Calcofluor white M2R fluorochrome (Sigma; 0.25 μg/mL in dH_2_O). For hemicelluloses staining, sections were incubated with LM10 (http://glycomics.ccrc.uga.edu/wall2/antibodies/antibodyHome.html). Sections were imaged using a microscope (Olympus BX-61, Japan) equipped with the following filter sets: 350/450 nm (ex/em) and 490/520 nm (ex/em) for visualizing Calcofluor white-stained cell walls and green emission of the FITC fluorochrome, respectively.

Scanning electron microscopy (SEM) was used to observe cell wall structures and the effects of pretreatment. Cross sections were obtained by dissecting transversely with razor blade by hand and the samples were attached using double-sided stick tapes. The samples were observed by SEM (PhenomtmPure FEI, USA) following the manual’s recommendations and images were captured digitally. For the effects of pretreatment, the biomass residues after pretreatment were dried at 50 °C to constant weight, and the surfaces of biomass samples were observed using SEM. Each sample was observed 10 times, and a representative image was used in this study.

#### Crude cellulose hydrolysis by β-1,4-exoglucanase (CBHI)

CBHI enzyme hydrolysis assay was performed using crude cellulose samples as described by Huang et al. [19]. Samples were incubated with CBHI (E.C. 3.2.1.91; Megazyme, USA) at 50 °C for a time course of reactions. After centrifugation, the supernatants were collected and treated with TFA, and *Myo*-inositol was added as the internal standard. The supernatants were then dried under vacuum to remove TFA. Distilled water and freshly prepared solution of sodium borohydride were added to each sample, incubated at 40 °C for 1 h, and the excess sodium borohydride was decomposed by adding acetic acid. 1-methylimidazole and the acetic anhydride were added and mixed well to perform an acetylation reaction. The excess acetic anhydride was decomposed by adding distilled water. Dichloromethane was added, mixed gently, and left standing for phase separation. The collected samples were analyzed using GC–MS (SHIMADZU GCMS-QP2010 Plus) as described above.

#### Measurement of cellulose accessibility and biomass porosity

Congo red stain was applied to estimate cellulosic surface area accessible for degrading cellulases as previously described by Wiman et al. [44]. 100 mg sample was treated with Congo red solution under increasing concentrations (0.25, 0.50, 0.75, 1.0, 1.5, 2.0 mg/mL) in 0.3 M phosphate buffer (pH 6.0) with 1.4 mM NaCl at 60 °C for 24 h with 200 rpm rotation speed. After centrifugation at 8000 g for 5 min, the absorbance of the supernatant was recorded at 498 nm. Adsorption of Congo red (Ae, mg/g) was calculated by Langmuir model using the following equation: Ae = (Ci − Ce) × *V*/(*M* × 1000). *V*, total volume (mL) at determination; *M*, initial weight of biomass (g). In this study, *V* was 10 mL, *M* was 0.1 g. Ci and Ce, Congo red concentrations (mg/L) before or after adsorption, calculated using standard curve from Congo red solutions at 20, 40, 60, 80, 100 and 120 mg/L concentrations.

Measurements of specific surface area, and mean pore radius were conducted using the multipurpose apparatus Micrometrics ASAP 2460 (USA) as described by Brunauer et al. [64], Liu et al. [65], and Li et al. [18]. The specific surface area was calculated by the Brunauer–Emmett–Teller (BET) method with the adsorption data at the relative pressure (*P*/*P0*) range of 0.05–0.3. The total pore volumes were measured at *P*/*P0* = 0.95. The average pore diameter was obtained using the iterative method of Barrett–Joyner–Halenda (BJH) and BET.

### Statistical analysis

Biological triplicate samples were collected for 5 plants of each transgenic line selection, and chemical analysis was performed in technical triplicates. The SPSS statistical software was used for data analysis. Statistical analysis was performed by Student’s *t* tests (two tail distribution and two samples with unequal variances) as *P < 0.05 and **P < 0.01.

## Supplementary information

**Additional file 1: Table S1.** Information of DET2 genes. Pe: *Populus euphratica*, Ptr: *P. trichocarpa*, Me: Manihot esculenta, Jc: *Jatropha curcas*, Ai: *Arachis ipaensis*, Vv: *Vitis vinifera*, Ha: *Helianthus annuus*, Pb: *Pyrus* x *bretschneideri*, At: *A. thaliana*, Cq: *Chenopodium quinoa*, Mn: *Morus notabilis*, Eg: *Eucalyptus grandis*, Gr: *Gossypium raimondii*, Gb: *Gossypium barbadense*, Gh: *Gossypium hirsutum*, Sl: *Solanum lycopersicum*, Sc: *Solanum chacoense*, Os: *Oryza sativa*, Zm: *Zea mays*, Sb: *Sorghum bicolor*. **Table S2.** Gene primers used for PCR amplification. **Table S3.** Total pectin and lignin contents (% biomass) in stems of transgenic lines and WT. All data are given as mean ± SD (*n* = 3, technical replicates). Statistical analyses were performed using Student’s *t* test as **P < 0.01 and *P < 0.05. **Table S4.** Wall polymer features in stems of transgenic lines and WT. Rha, Rhamnose; Fuc, Fucose; Ara, Arabinose; Xyl, Xylose; Man, Mannose; Gal, Galactose. **Table S5.** Characteristic peaks of the FTIR spectra in biomass residues as referred from previous studies. **Table S6.** Comparison of bioethanol yields obtained in the transgenic poplar plant and other woody plants. **Fig. S1** Multiple sequence alignment and phylogenetic analysis of DET2. (a) The phylogenetic relationship of *PtoDET2* with other DET2 proteins. (b) Sequence alignments of *PtoDET2*. The GenBank accession numbers of DET2s were listed in Table S1. **Fig. S2** Generation of transgenic poplars. (a) Diagram of the *PtoDET2*-*OE* vector. (b) The Hyg levels in the *PtoDET2*-*OE* lines. (c) The expression levels of *PtoDET2* in the *PtoDET2*-*OE* lines. The poplar *ubiquitin* gene was used as an internal control. (d) Diagram of three CRISPR/Cas9 target sites of *PtoDET2*. T1, T2 and T3 indicate the positions of sgRNA-targeted sites. (e) Determination of the mutations in the coding region of *PtoDET2* generated by the CRISPR/Cas9 system. The text on the right summarizes mutation details in two independent CRISPR/Cas9-generated lines (L11 and L17). Primers are listed in Table S2. **Fig. S3** Expression of cell differentiation, expansion and wall biosynthetic genes in *PtoDET2* transgenic plants. (a) Cell differentiation genes; (b) cell expansion genes; (c) cellulose biosynthetic genes; (d) hemicellulose biosynthetic genes. Primers are listed in Table S2. The poplar *ubiquitin* gene was used as an internal control. All data are given as mean ± SD from three biological repeats. Statistical analyses were performed using Student’s *t* test as **P < 0.01. **Fig. S4** Hexoses released from enzymatic hydrolysis after various chemical pretreatments. (a) Hexose yields released from enzymatic (mixed-cellulase) hydrolysis after pretreatments with H_2_SO_4_, (b) NaOH, (c) CaO or (d) Na_2_S + Na_2_CO_3_. All data are given as mean ± SD of three technical repeats. **Fig. S5** Expression of *PtoGH9* genes in transgenic *PtoDET2* plants. Primers are listed in Table S2 available as Supplementary Data. The poplar *ubiquitin* gene was used as an internal control. All data are given as mean ± SD from three technical repeats. Statistical analyses were performed using Student’s *t* test as **P < 0.01. **Fig. S6** Mass balance analysis for bioethanol production during biomass process with green liquor in transgenic poplar lines and WT. **Fig. S7** Fourier transform infrared spectra (FTIR) profiling in transgenic poplar lines and WT. Black line as raw material (R) and red line as biomass residue from Na_2_S + Na_2_CO_3_ pretreatment (P). Characteristic peaks of the FTIR spectra were referred in Table S5. **Fig. S8** A hypothetical model to demonstrate an integrated approach effective for maximum bioethanol production in lignocellulose-improved transgenic poplar plants overproducing BRs.

## Data Availability

All data generated or analyzed during this study are included in this published article and its additional file. Plant materials used in this study are available from corresponding author, Keming Luo (kemingl@swu.edu.cn).

## Abbreviations

DET2: DEETIOLATED2
BR: brassinosteroid
CrI: cellulose crystallinity index
DP: degree of polymerization
Ara: arabinose
Xyl: xylose
SEM: scanning electron microscopy
CBH: cellobiohydrolase
Ph: phloem
C: cambium
Xy: xylem
Xf: xylem fiber cells
Xv: xylem vessel cells
P: pith
Ep: epidermis
GH: glycoside hydrolase
FTIR: Fourier transform infrared
