# Supplementary material for: Brassinosteroid overproduction improves lignocellulose quantity and quality to maximize bioethanol yield under green-like biomass process in transgenic poplar
Source: Biotechnol Biofuels. 2020 Jan 18;13:9. doi: 10.1186/s13068-020-1652-z (PMC6969456; doi:10.1186/s13068-020-1652-z)
Supplement: Supplementary file 1 — Additional file 1: Table S1. Information of DET2 genes. Pe: Populus euphratica, Ptr: P. trichocarpa, Me: Manihot esculenta, Jc: Jatropha curcas, Ai: Arachis ipaensis, Vv: Vitis vinifera, Ha: Helianthus annuus, Pb: Pyrus x bretschneideri, At: A. thaliana, Cq: Chenopodium quinoa, Mn: Morus notabilis, Eg: Eucalyptus grandis, Gr: Gossypium raimondii, Gb: Gossypium barbadense, Gh: Gossypium hirsutum, Sl: Solanum lycopersicum, Sc: Solanum chacoense, Os: Oryza sativa, Zm: Zea mays, Sb: Sorghum bicolor. Table S2. Gene primers used for PCR amplification. Table S3. Total pectin and lignin contents (% biomass) in stems of transgenic lines and WT. All data are given as mean ± SD (n = 3, technical replicates). Statistical analyses were performed using Student’s t test as **P < 0.01 and *P < 0.05. Table S4. Wall polymer features in stems of transgenic lines and WT. Rha, Rhamnose; Fuc, Fucose; Ara, Arabinose; Xyl, Xylose; Man, Mannose; Gal, Galactose. Table S5. Characteristic peaks of the FTIR spectra in biomass residues as referred from previous studies. Table S6. Comparison of bioethanol yields obtained in the transgenic poplar plant and other woody plants. Fig. S1 Multiple sequence alignment and phylogenetic analysis of DET2. (a) The phylogenetic relationship of PtoDET2 with other DET2 proteins. (b) Sequence alignments of PtoDET2. The GenBank accession numbers of DET2s were listed in Table S1. Fig. S2 Generation of transgenic poplars. (a) Diagram of the PtoDET2-OE vector. (b) The Hyg levels in the PtoDET2-OE lines. (c) The expression levels of PtoDET2 in the PtoDET2-OE lines. The poplar ubiquitin gene was used as an internal control. (d) Diagram of three CRISPR/Cas9 target sites of PtoDET2. T1, T2 and T3 indicate the positions of sgRNA-targeted sites. (e) Determination of the mutations in the coding region of PtoDET2 generated by the CRISPR/Cas9 system. The text on the right summarizes mutation details in two independent CRISPR/Cas9-generated lines (L11 and L17). Primers are li [file 13068_2020_1652_MOESM1_ESM.pdf]

Table S1. Information on *DET2* genes.

No.	Genes	Accessions	No.	Genes	Accessions
1	AtDET2	AT2G38050.1	11	MeDET2	XP_021619903.1
2	VvDET2	XP_002277900.1	12	JcDET2	XP_012087924.1
3	OsDET2	LOC_Os01g63260.1	13	PbDET2	XP_009370214.1
4	GhDET2	AAN28012.1	14	PeDET2	XP_011009141.1
5	GbDET2	AIY32624.1	15	PtrDET2	XP_006382738.1
6	GrDET2	XP_012458951.1	16	MnDET2	XP_010101824.1
7	ZmDET2	NP_001149816.1	17	HaDET2	XP_021998413.1
8	SlDET2	NP_001234040.1	18	CqDET2	XP_021741624.1
9	ScDET2	ABD96045.1 5	19	AiDET2	XP_016198496.1
10	SbDET2	AEW49992.1	20	EgDET2	XP_010053477.1

Pe: *Populus euphratica*, Ptr: *P. trichocarpa*, Me: Manihot esculenta, Jc: *Jatropha curcas*, Ai: *Arachis ipaensis*, Vv: *Vitis vinifera*, Ha: *Helianthus annuus*, Pb: *Pyrus* x *bretschneideri*, At: *A. thaliana*, Cq: *Chenopodium quinoa*, Mn: *Morus notabilis*, Eg: *Eucalyptus grandis*, Gr: *Gossypium raimondii*, Gb: *Gossypium barbadense*, Gh: *Gossypium hirsutum*, Sl: *Solanum lycopersicum*, Sc: *Solanum chacoense*, Os: *Oryza sativa*, Zm: *Zea mays*, Sb: *Sorghum bicolor*.

Table S2. Gene-specific primers used for PCR amplification.

Genes	Purpose	Forward primer (5'-3')	Reverse primer (5'-3')
PtoDET2	Gene cloning	ATGGCCCTATTAGATCAGAG	AGCTTCAACACAGAAAAGG
	Cas9-T1	GTCATCCTATCCATTTACCTCAT	AAACATGAGGTAAATGGATAGGA
	Cas9-T2	ATTGCACCTGCATCTACCCACTT	AAACAAGTGGGTAGATGCAGGTG
	Cas9-T3	ATTGATTGTGGAGTGGCTTGGAT	AAACATCCAAGCCACTCCACAAT
	Q-PCR	GGTCCTACAATCTCTCCACCCTTG	AGGAGGAGTGTGAGCCAAAGAG
Hyg	PCR	ATCGGACGATTGCGTCGCATC	GTGTCACGTTGCAAGACCTG
CPD	Q-PCR	TGCCCAGGATATGAGCTTGC	TCGCTTCTGTGTCCGTGTAG
ROT3.1	Q-PCR	GCTGCTGGCTACACTTCTCA	GGTGACCTCAAGAAACCACCA
CYP84A2	Q-PCR	AGCAGTTCATGGCTCCACTC	AGTGCCAGGAAGGTCAATCG
LBD38	Q-PCR	GAAGAGTCTGAAACCACAACAC	CTTGTTCAGTCGCTCATGTATG
CLE14	Q-PCR	AGCTCATTTTGTTTCTCACGAG	TTTGGCTAAGCTTGAAATCACG
EXPA5	Q-PCR	TACCACCGTCTATCTCCACTGTC	GGCTAACCATCGTTTGCG
EXPA12	Q-PCR	TGACAAGGAGTATCAGTATTTTTCG	AGGTGTATCTATCAGGCGAGGA
CESA2B	Q-PCR	AGGTTAAGATGGAGCGG	ACGAGGTTGATGATCAAGCC
CESA3A	Q-PCR	CCAGGCAACCACTATGGAGAA	ATTAGGCTCACCCTCACGCT
GT8D	Q-PCR	GAATTTATGGACGAAGTCAAGAACAC	GCTGCTTCGGTATGCTACTTGATGCT
GT43B	Q-PCR	CCAGCTCCACCAAGCTCTAA	ATGATCCAACTCTGCTTCGGG
GH9A1	Q-PCR	CCGCTGTCCACCATTCATAA	CGGGCTGTGTTACTCTCTC
GH9A2	Q-PCR	GCTGTGCCTCGCCATCGTCACAA	GAGCAAGGGTGTAGTTATCAGCG
UBQ	Q-PCR	GTTGATTTTTGCTGGGAAGC	GATCTTGGCCTTCACGTTGT

Table S3. Total pectin and lignin contents (% biomass) in stems of transgenic lines and WT.

Samples	Pectin (% biomass)		Total Lignin (% biomass)
WT	3.21±0.05		20.61±0.21
PtoDET2-OE-L1	3.65±0.06**	+13%	20.43±0.10
PtoDET2-OE-L5	3.50±0.05**	+9%	20.10±0.42
PtoDET2-KO-L11	2.93±0.11**	-9%	19.90±0.32
PtoDET2-KO-L17	2.85±0.08**	-11%	20.01±0.25

All data are given as means ± SD (n = 3). Statistical analyses were performed using Student's *t* test as ***P* < 0.01 and **P* < 0.05.

Table S4. Cell wall features in the raw materials.

Samples	Monosaccharides of hemicelluloses (% total)						Lignin monolignols (% total)			
	Rha	Fuc	Ara	Xyl	Man	Gal	H	G	S	S/G
WT	0.39	0.04	2.44	90.19	2.81	4.14	0.13	43.05	56.82	1.32
PtoDET2-OE-L1	0.42	0.09	**3.84**	**88.11**	**2.25**	**5.29**	0.13	43.56	56.32	1.29
PtoDET2-OE-L5	0.41	0.06	**3.10**	**89.45**	**2.26**	**4.72**	0.14	43.45	56.40	1.30
PtoDET2-KO-L11	0.41	0.04	**2.05**	**90.50**	**3.01**	**3.99**	0.12	42.77	57.11	1.34
PtoDET2-KO-L17	0.42	0.05	**2.05**	**90.46**	**3.02**	**4.00**	0.15	43.45	56.40	1.30

Rha, Rhamnose; Fuc, Fucose; Ara, Arabinose; Xyl, Xylose; Man, Mannose; Gal, Galactose.

Table S5. Characteristic peaks of the FTIR spectra in biomass residues.

Reported wave number (cm^{-1})	Observed wave number (cm^{-1})	Functional group	Assignment
898	898	C—H vibration	Cellulose
**1051**	**1052**	**C—O—C ring skeletal vibration**	**Hemicelluloses**
1163	1160	C—O—C asymmetric stretching	Cellulose
**1247**	**1244**	**C—O—C stretching of aryl-alkyl ether**	**Lignin**
1373	1367	C—H_2 scissoring	Cellulose
1430	1430	C—H_2 bending	Cellulose
1460	1460	C—H_3 asymmetric bending	Lignin
**1515**	**1508**	**C=C stretching of the aromatic ring**	**Lignin**
**1603**	**1615**	**C=C stretching**	**Lignin**
**1735**	**1736**	**C=O stretching of acetyl or carboxylic acid**	**Hemicelluloses & lignin**

Table S6. Comparison of bioethanol yields obtained in the transgenic poplar plant and other woody plants.

	Pretreatments	Ethanol yield (% biomass)	Reference
**WT (poplar)**	**$Na_2S+Na_2CO_3$, 150 ºC, 20 min**	**11.79%**	**This study**
**DET2-OE-L1 (poplar)**	**$Na_2S+Na_2CO_3$, 150 ºC, 20 min**	**15.68%**	
WT (poplar)	Hot water, 180 ºC, 20 min	4.1%	Biswal et al., 2018
GAUT4-KD (poplar)	Hot water, 180 ºC, 20 min	6.7%	Biswal et al., 2018
COMT3-TG (poplar)	Hot water, 180 ºC, 20 min	6.1%	Biswal et al., 2018
WT (poplar)	1% $Ca(OH)_2$, 121 ºC, 6 h	12.1% (48h)	Cai et al., 2016
MOMT4-OE (poplar)	1% $Ca(OH)_2$, 121 ºC, 6 h	14.3% (48h)	Cai et al., 2016
Eucalyptus globulus	Steam explosion, 190 ºC, 10 min, 1.5% H_2SO_4	10.75-11.52%	Ko et al., 2012
	Steam explosion, 195 ºC, 34 min	10.18%	Romaní et al., 2013
Olive tree	Steam explosion, 230 °C, 5 min, 2% H_2SO_4	7.2%	Cara et al., 2008

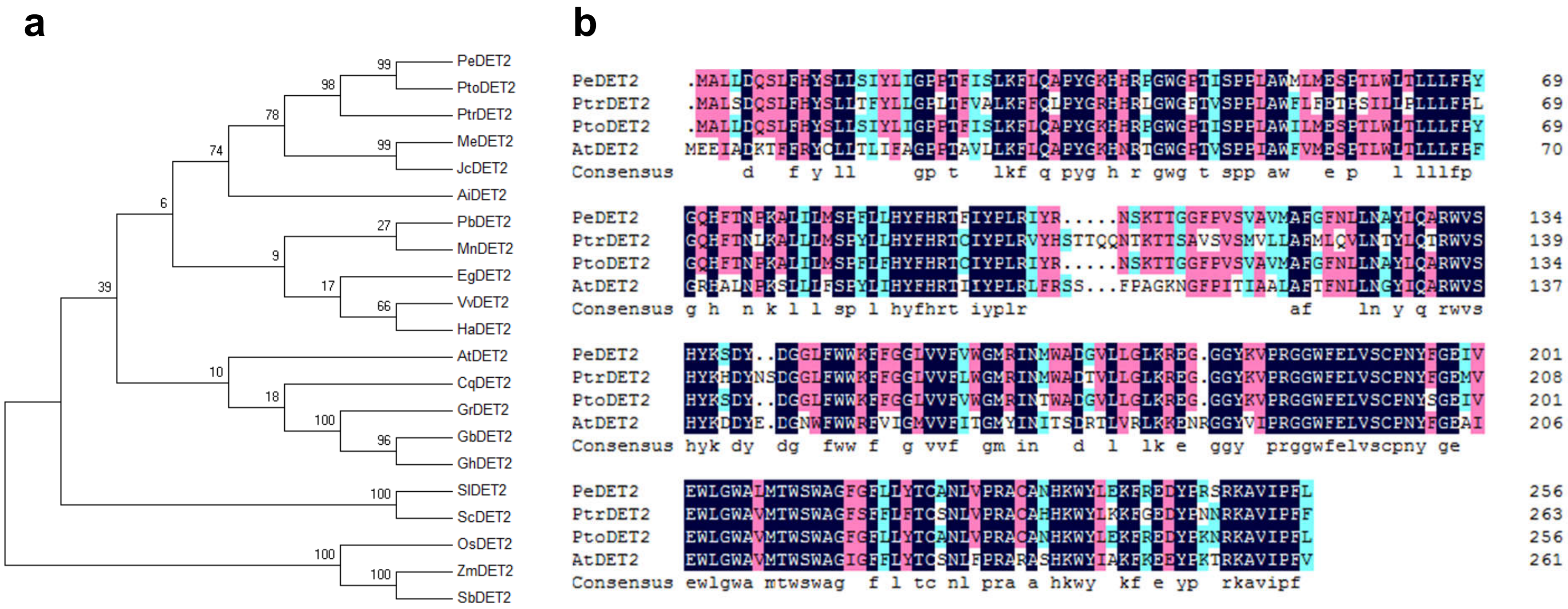

Fig S1. Multiple sequence alignment and phylogenetic analysis of DET2. (a) The phylogenetic relationship of PtoDET2 with other DET2 proteins. (b) Sequence alignments of PtoDET2. The GenBank accession numbers of DET2s were listed in Table S1.

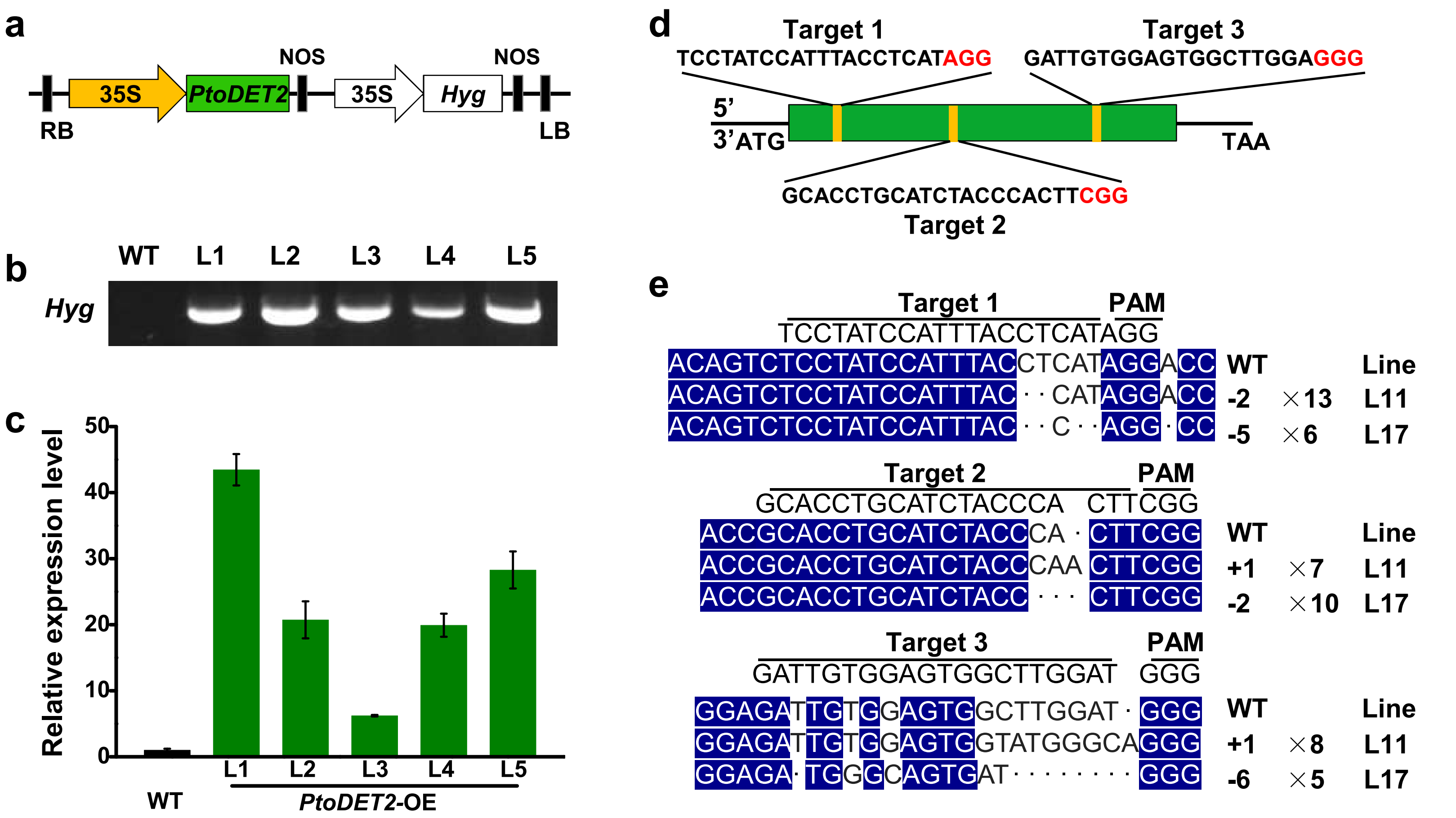

Fig S2. Generation of transgenic poplars. (a) Diagram of the *PtoDET2-OE* vector. (b) The Hyg levels in the *PtoDET2-OE* lines. (c) The expression levels of *PtoDET2* in the *PtoDET2-OE* lines. (d) Diagram of three CRISPR/Cas9 target sites of *PtoDET2*. T1, T2 and T3 indicate the positions of sgRNA-targeted sites. (e) Determination of the mutations in the coding region of *PtoDET2* generated by the CRISPR/Cas9 system. The text on the right summarizes mutation details in two independent CRISPR/Cas9-generated lines (L11 and L17). Primers are listed in Table S2. The poplar *ubiquitin* gene was used as an internal control.

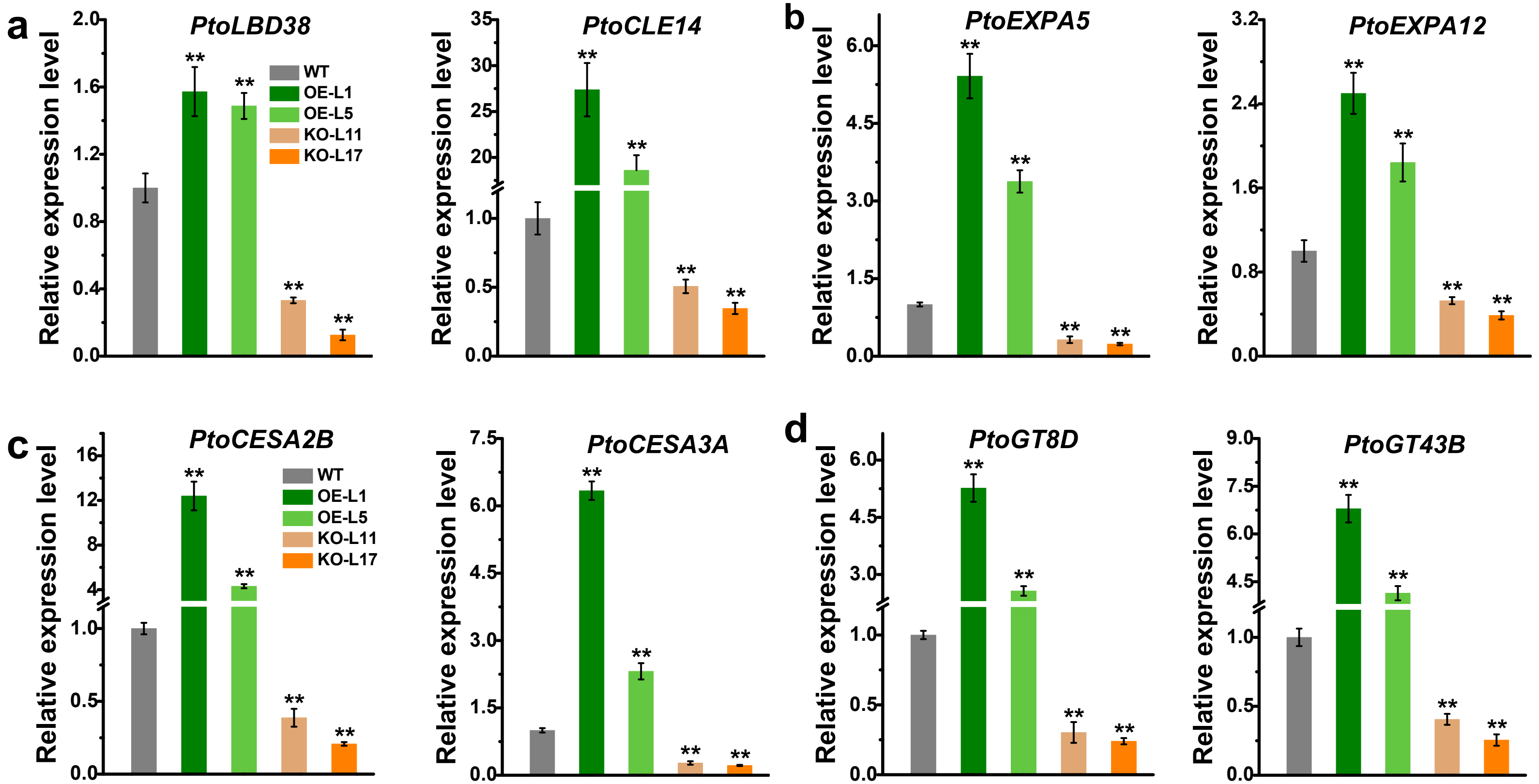

Fig S3. Expression of cell differentiation, expansion and wall biosynthetic genes in *PtoDET2* transgenic plants. (a) Cell differentiation genes; (b) Cell expansion genes; (c) Cellulose biosynthetic genes; (d) Hemicellulose biosynthetic genes. Primers are listed in Table S2. The poplar *ubiquitin* gene was used as an internal control. All data are given as means ± SD from three biological repeats. Statistical analyses were performed using Student's *t* test as $**P < 0.01$.

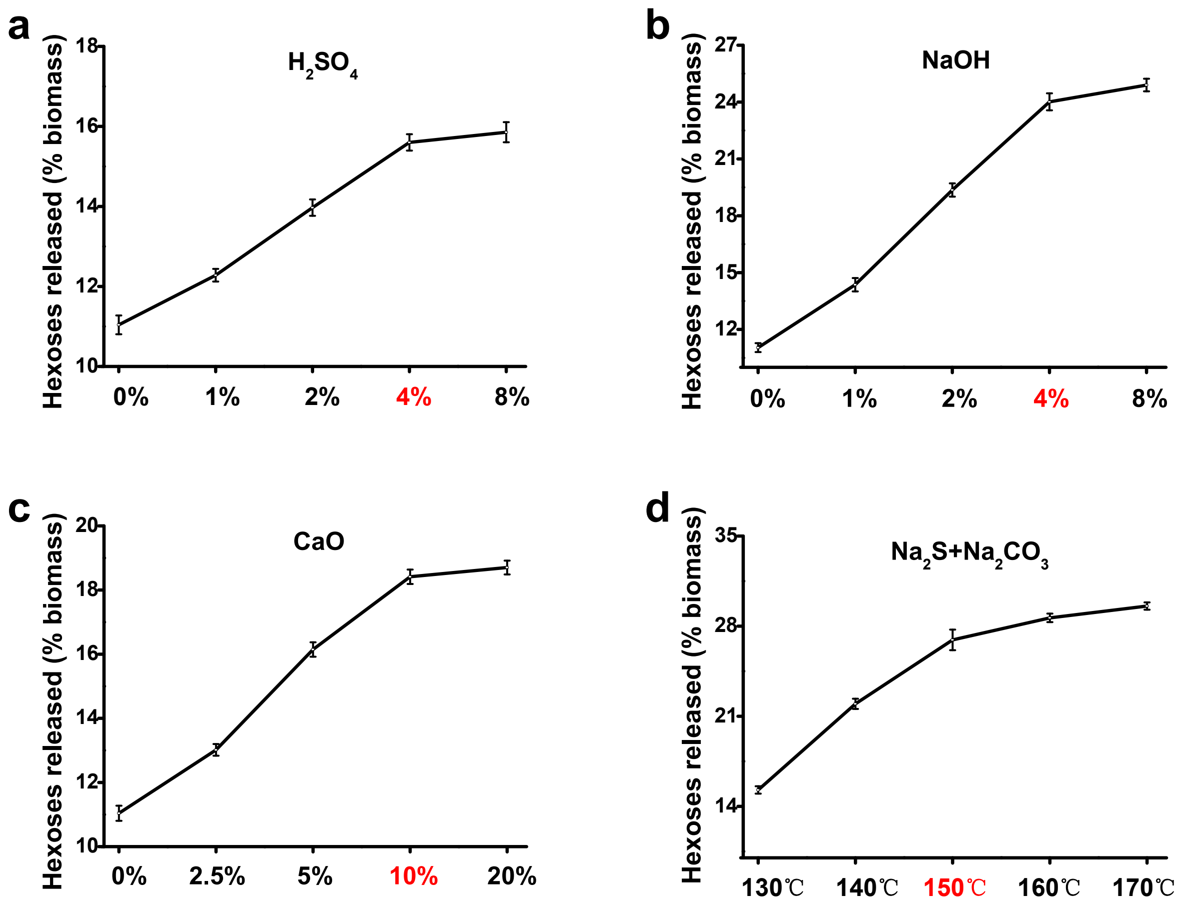

Fig S4. Hexoses released from enzymatic hydrolysis after various chemical pretreatments. (a) Hexose yields released from enzymatic (mixed-cellulase) hydrolysis after pretreatments with H_2SO_4, (b) NaOH, (c) CaO or (d) Na_2S+Na_2CO_3. All data are given as means ± SD from three technical repeats.

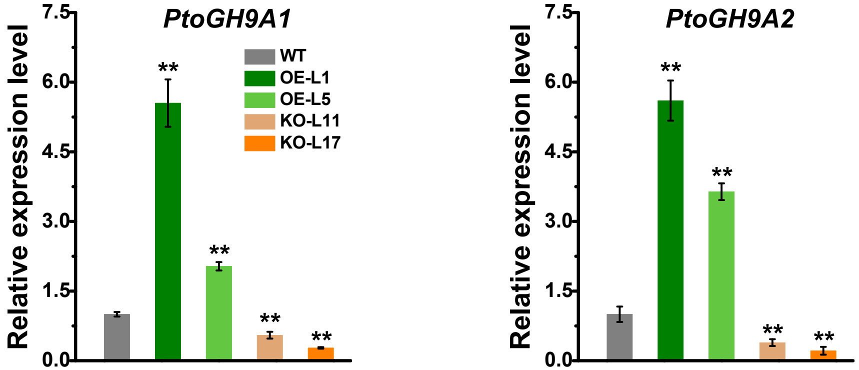

Fig S5. Expression of *PtoGH9* genes in transgenic *PtoDET2* plants. Primers are listed in Table S2. The poplar *polyubiquitin* gene was used as an internal control. All data are given as means ± SD from three technical repeats. Statistical analyses were performed using Student's *t* test as $^{**}P < 0.01$.

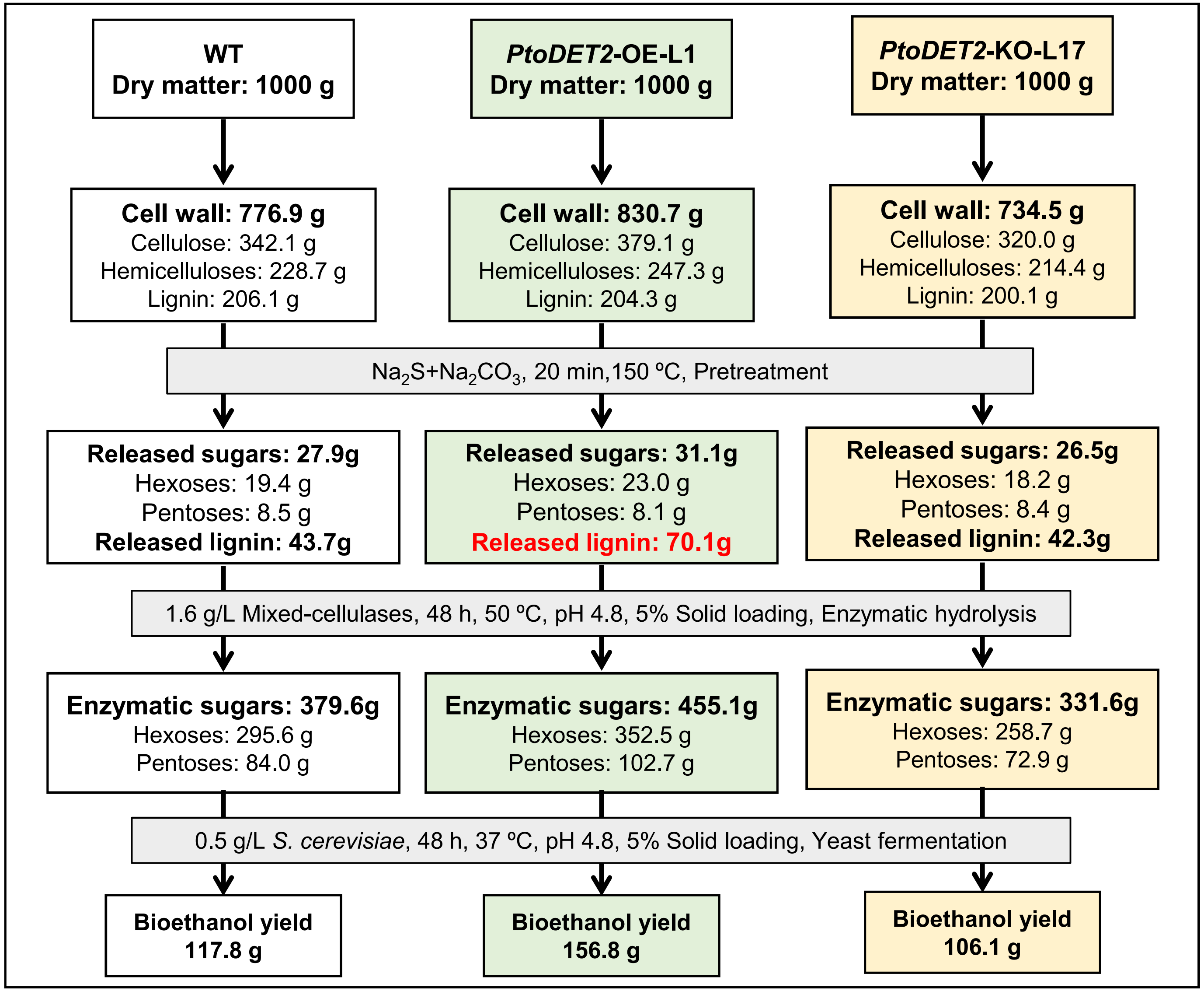

Fig S6. Mass balance analysis for bioethanol production during biomass process with green liquor in transgenic poplar lines and WT.

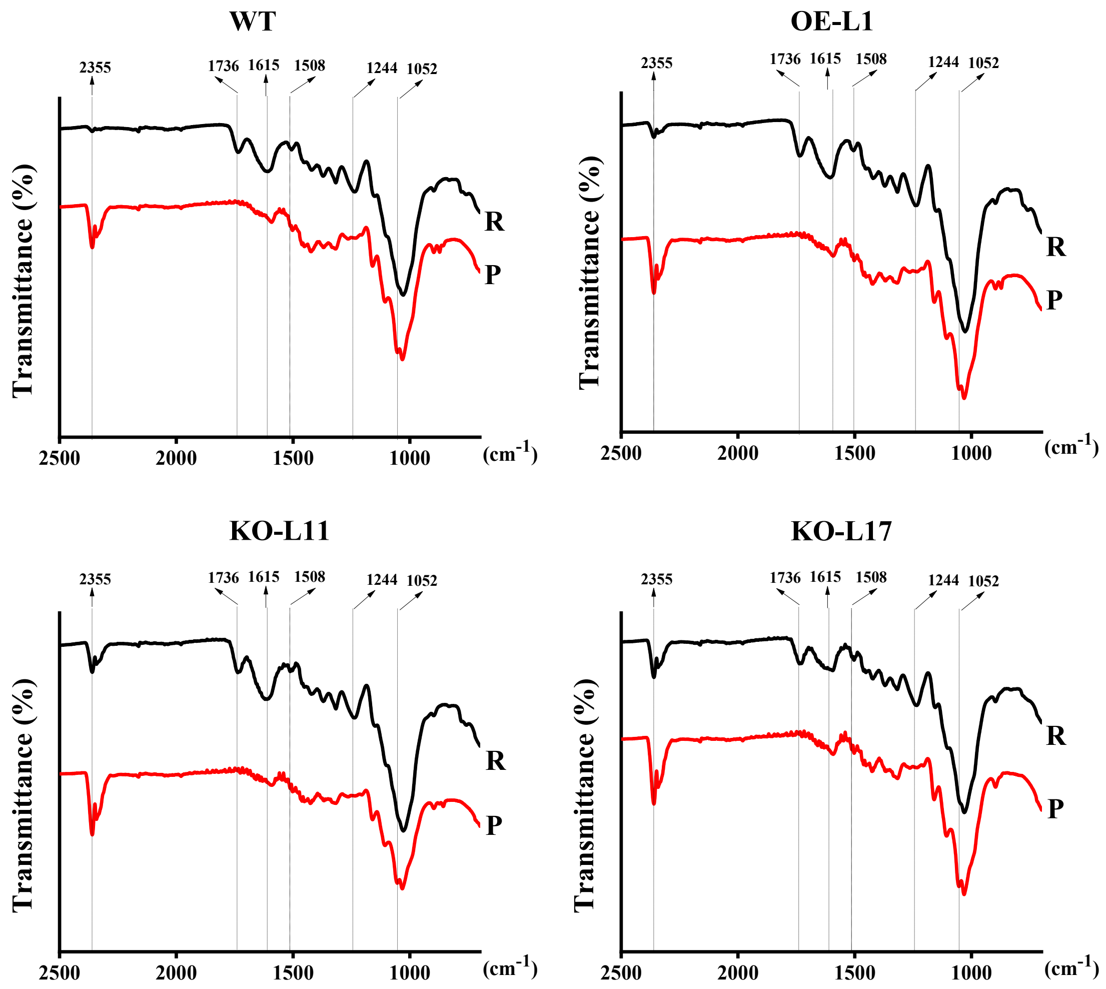

Fig S7. Fourier transform infrared spectra (FTIR) profiling in transgenic poplar lines and WT. Black line as raw material (R) and red line as biomass residue from Na_2S+Na_2CO_3 pretreatment (P). Characteristic peaks of the FTIR spectra were referred in Table S5.

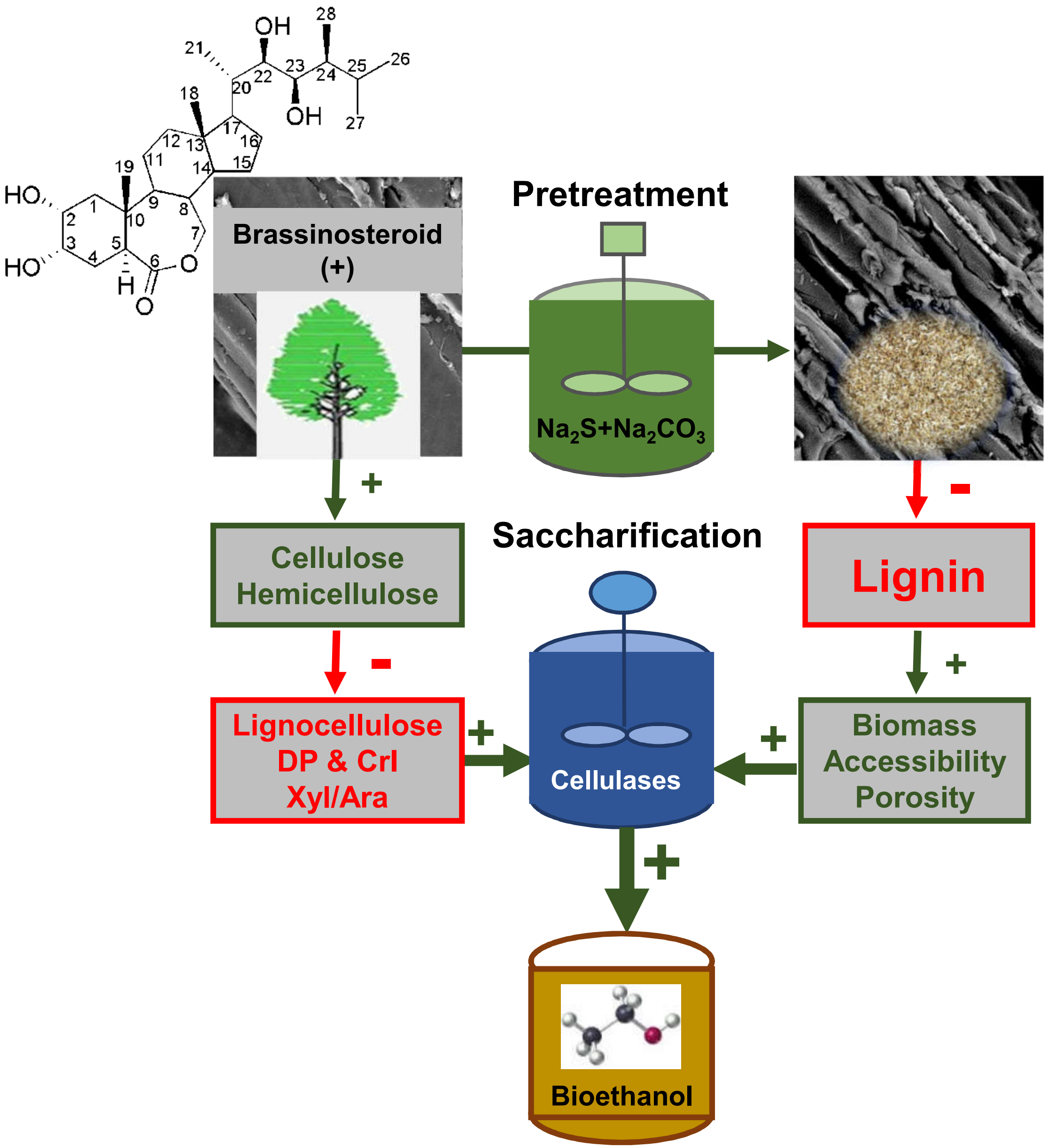

Fig S8. A hypothetical model to demonstrate an integrated approach effective for maximum bioethanol production in lignocellulose-improved transgenic poplar plants overproducing BRs.
